# Targeting ROS-sensing Nrf2 potentiates anti-tumor immunity of intratumoral CD8^+^ T and CAR-T cells

**DOI:** 10.1016/j.ymthe.2024.08.019

**Published:** 2024-08-22

**Authors:** Yuna Jo, Ju A. Shim, Jin Woo Jeong, Hyori Kim, So Min Lee, Juhee Jeong, Segi Kim, Sun-Kyoung Im, Donghoon Choi, Byung Ha Lee, Yun Hak Kim, Chi Dae Kim, Chan Hyuk Kim, Changwan Hong

**Affiliations:** 1 Department of Anatomy, Pusan National University School of Medicine, Yangsan 50612, Republic of Korea; 2 Department of Convergence Medical Science, Pusan National University School of Medicine, Yangsan 50612, Republic of Korea; 3 PNU GRAND Convergence Medical Science Education Research Center, Pusan National University School of Medicine, Yangsan 50612, Republic of Korea; 4 Department of Anatomy and Cell Biology, Department of Biomedical Sciences, Seoul National University College of Medicine, Seoul 03080, Republic of Korea; 5 Department of Biological Sciences, Korea Advanced Institute of Science and Technology, Daejeon 34141, Republic of Korea; 6 NeoImmunetech, Co., Ltd., Pohang 37666, Republic of Korea; 7 NeoImmunetech, Inc., Rockville, MD 20850, USA; 8 Department of Pharmacology, Pusan National University School of Medicine, Yangsan 50612, Republic of Korea; 9 School of Transdisciplinary Innovations and College of Pharmacy, Seoul National University, Seoul 08826, Republic of Korea

**Keywords:** Nrf2, reactive oxygen species, T cell immunotherapy, anti-tumor immune responses, tumor microenvironment, CAR T cells

## Abstract

Cytotoxic T lymphocytes (CTLs) play a crucial role in cancer rejection. However, CTLs encounter dysfunction and exhaustion in the immunosuppressive tumor microenvironment (TME). Although the reactive oxygen species (ROS)-rich TME attenuates CTL function, the underlying molecular mechanism remains poorly understood. The nuclear factor erythroid 2-related 2 (Nrf2) is the ROS-responsible factor implicated in increasing susceptibility to cancer progression. Therefore, we examined how Nrf2 is involved in anti-tumor responses of CD8^+^ T and chimeric antigen receptor (CAR) T cells in the ROS-rich TME. Here, we demonstrated that tumor growth in *Nrf2*^−/−^ mice was significantly controlled and was reversed by T cell depletion and further confirmed that Nrf2 deficiency in T cells promotes anti-tumor responses using an adoptive transfer model of antigen-specific CD8^+^ T cells. Nrf2-deficient CTLs are resistant to ROS, and their effector functions are sustained in the TME. Furthermore, Nrf2 knockdown in human CAR-T cells enhanced the survival and function of intratumoral CAR-T cells in a solid tumor xenograft model and effectively controlled tumor growth. ROS-sensing Nrf2 inhibits the anti-tumor T cell responses, indicating that Nrf2 may be a potential target for T cell immunotherapy strategies against solid tumors.

## Graphical abstract

**Figure:**
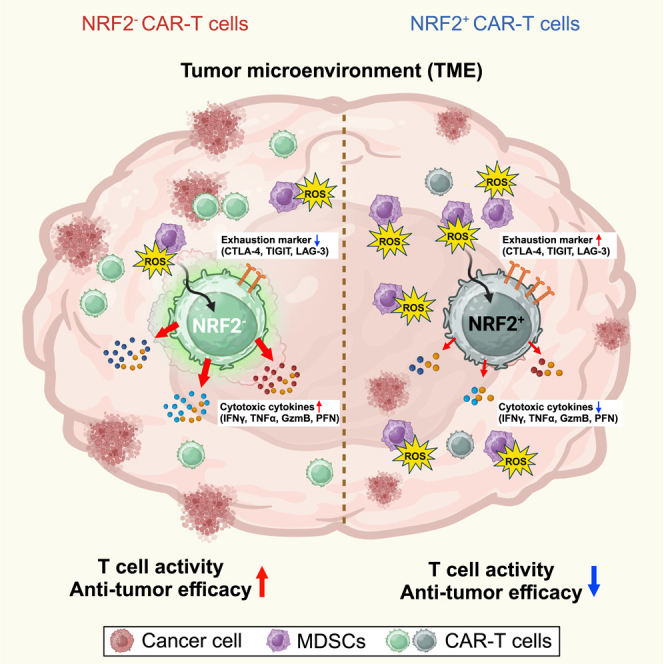

## Teaser

Hong and colleagues found that Nrf2 deficiency in CD8^+^ T and CAR T cells enhances anti-tumor responses in ROS-rich tumor microenvironments. Nrf2-deficient CTLs resist ROS and maintain effector functions. Nrf2 knockdown in human CAR-T cells improves survival and function, controlling tumor growth. Thus, targeting Nrf2 may enhance T cell immunotherapy against solid tumors.

## Introduction

CD8^+^ cytotoxic T lymphocytes (CTLs) play a pivotal role in protective immune functions by recognizing and killing cancer cells.^1^^,^^2^ However, CTL responses are often dampened in solid tumors by immunosuppressive factors and cells, including inhibitory cytokines/molecules, Foxp3^+^CD4^+^ T cells (Tregs), tumor-associated macrophages (TAMs), and myeloid-derived suppressor cells (MDSCs).^3^^,^^4^^,^^5^^,^^6^ MDSCs induce immunosuppression by producing reactive oxygen species (ROS) that inhibit CD8^+^ T cell activation and proliferation.^7^^,^^8^^,^^9^ The distribution of MDSCs in tumors is correlated with immune suppression and the poor reactivity of T cells to immune checkpoint inhibition and anticancer vaccinations.^10^^,^^11^^,^^12^ Although ROS in tumors are critical negative regulators of T cell receptor (TCR)-major histocompatibility complex interactions,^13^^,^^14^ T cell-intrinsic transcription factors (TFs) may also influence anti-tumor activity in the ROS-rich tumor microenvironment (TME).

The TF nuclear factor erythroid 2-related 2 (Nrf2) functions as a critical intracellular sensor and protector of oxidative stress (OS).^15^^,^^16^ Nrf2 expression is regulated through the Kelch-like ECH-associated protein 1 (Keap1) using the ubiquitin-proteasome pathway.^17^ Keap1 degrades Nrf2 proteins under homeostatic conditions, whereas Nrf2 degradation under conditions of OS, such as high levels of ROS, is inhibited via conformational changes in thiol residues in Keap1, activating the antioxidant functions of Nrf2 instead.^17^^,^^18^ In the context of cancer research, the role of Nrf2 has been elucidated mainly in tumor cells^19^^,^^20^^,^^21^^,^^22^ and MDSCs.^23^^,^^24^ However, little is known about its role in T cells, the important cellular components of the TME. Given that T cells are exposed to the ROS-rich TME, it is plausible to infer its relevance in T cells. Recent bioinformatics analyses using an activation-independent exhaustion program (Mel75 exhaustion program) in tumor-infiltrating (TI) CD8^+^ T cells have demonstrated that NFE2L2 (Nrf2) in high-exhaustion cells is highly associated (*p* < 0.001 by permutation test) with core exhaustion signature genes in human patients.^25^^,^^26^^,^^27^ In addition, an association between Nrf2 expression induced by ROS and CTL-mediated anti-tumor immune responses has been reported.^28^^,^^29^ However, given the shortage of data on direct causation and *in vivo* analysis of the role of Nrf2 in CD8^+^ T cell-mediated anti-tumor responses has remained unclear.

Here, we aimed to determine Nrf2 expression in TI T cells and investigate its relationship with the effector function of CD8^+^ T cells. To this end, we found that Nrf2 expression inversely relates to effector function of CD8^+^ T cells and is highly upregulated in TI T cells. In solid tumor models, genetic disruption of Nrf2 proteins in effector T cells effectively controls tumor growth and promotes the efficacy of adoptive cell therapy (ACT). Anti-tumor responses of Nrf2-deficient CD8^+^ T cells notably sustain ROS-rich TMEs. Furthermore, we developed Nrf2-knockdown (KD) chimeric antigen receptor (CAR) T cells based on CD19 and found potent efficacy against preclinical solid tumor models. These findings could help elucidate the role of Nrf2 as a key regulator of CTL activity in the immunosuppressive TME and its potential as a target to increase the efficacy of T cell-based ACT against solid tumors.

## Results

### Anti-tumor responses and Nrf2 expression in TI T cells

**Figure 1:**
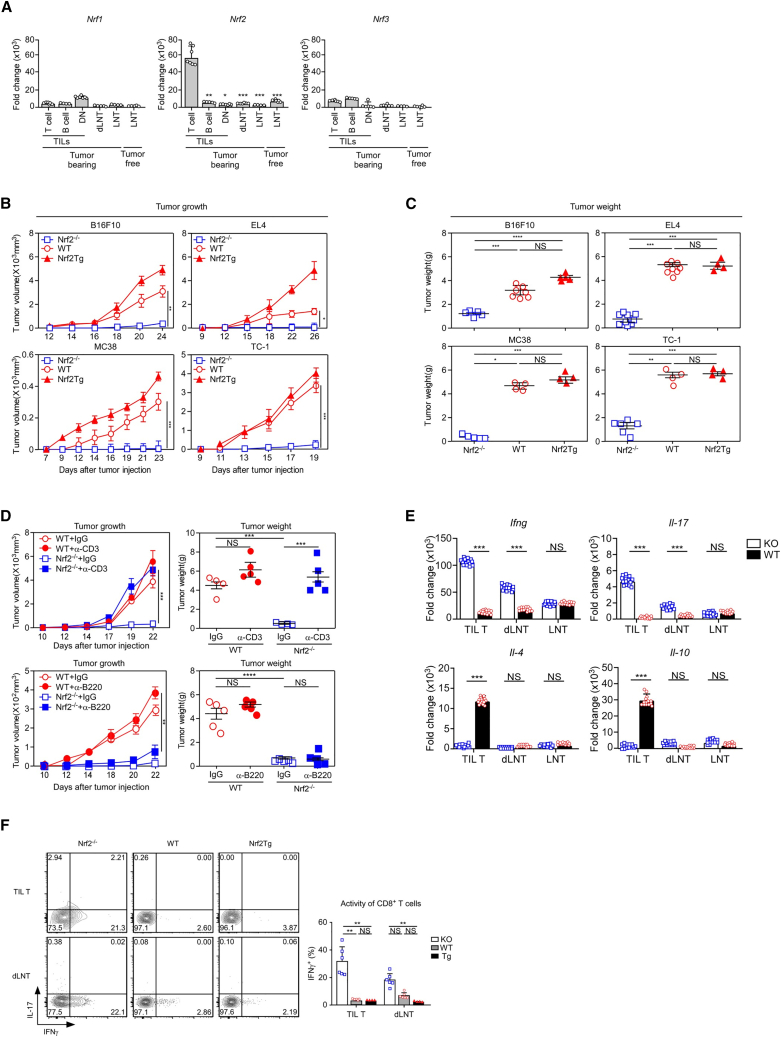
Nrf2 deficiency enhances anti-tumor activity *in vivo* (A) *Nrf1*, *Nrf2*, and *Nrf3* mRNA expression in T cells, B cells, and DN cells in TILs and in draining lymph node T (dLN T) and LN T cells from tumor-bearing mice. WT LN T cells are included as a control. The results represent the summary of three independent experiments (*n* ≥ 4 mice/group). The fold change of mRNA expression is 2^−ΔΔCt^ × 1,000 (ΔΔC_t_ = ΔC_t_ of the target gene − ΔC_t_ of the reference gene). (B and C) *Nrf2*^−/−^, WT, and Nrf2Tg mice are injected s.c. with B16F10 melanoma cells (three independent experiments, *n* ≥ 4 mice/group), EL4 lymphoma cells (four independent experiments, *n* ≥ 4 mice/group), MC38 colon carcinoma cells (two independent experiments, *n* ≥ 4 mice/group), or TC-1 lung carcinoma cells (one independent experiments *n* ≥ 4 mice/group). (B) Tumor growth is monitored every 2–3 days. (C) Tumor weight is measured at the end of the experiments. (D) Tumor growth and weight comparison between *Nrf2*^−/−^ and WT mice (*n* ≥ 4 mice/group) following s.c. injection of EL4 cells. Mice receive intraperitoneal injections of either α-CD3 (top) or α-B220 (bottom) antibodies or isotype control IgG once every 5 days. Results represent the summary of error of the mean of three independent experiments (∗*p* < 0.05, ∗∗*p* < 0.01, ∗∗∗*p* < 0.001, ∗∗∗∗*p* < 0.0001; NS, not significant). (E) RT-qPCR analysis of inflammatory cytokine genes in TIL T cells, dLN T cells, and LN T cells from the WT and *Nrf2*^−/−^ mice injected s.c. with B16F10 cells (*n* = 14, mice/group). The expression of the target genes is normalized to that of *Rpl13*. Data show the means ± SEM of four independent experiments (∗*p* < 0.05, ∗∗*p* < 0.01, ∗∗∗*p* < 0.001, ∗∗∗∗*p* < 0.0001). (F) IFNγ and IL-17 expression in TIL T and dLN T cells from *Nrf2*^−/−^, WT, and Nrf2Tg tumor-bearing mice. TIL T and dLN T cells were stimulated with PMA/ionomycin and assessed for IFNγ and IL-17 expression by intracellular staining. The IFNγ vs. IL-17 profile is representative of five independent experiments (left). The bar graph represents the percentage of IFNγ-producing T cells (right). Error bars depict the mean ± SEM of five independent experiments (∗*p* < 0.05, ∗∗*p* < 0.01, ∗∗∗*p* < 0.001).

According to a previous study,^26^ it has been suggested that the tumor-specific OS-related gene *NFE2L2* (*Nrf2*) is strongly associated with highly exhausted TI T cells (Figures S1A and S1B). Therefore, we examined the expression of the NFE2L family in TI cells and found that *Nrf2* expression was notably higher than that of other families (Figure 1A). Moreover, we found that it was significantly increased only in TI T cells compared with other immune cell populations (Figure 1A, center). Next, to assess whether Nrf2 expression affects the anti-tumor T cell response, we used Nrf2-deficient (*Nrf2*^−/−^) and Nrf2-transgenic (Tg) mice, in which Nrf2 was overexpressed under the control of the human CD2 (hCD2) promoter (Figures S2A–S2C). T cell development and homeostasis in *Nrf2*^−/−^ and Tg mice were comparable with those in wild-type (WT) mice (Figure S2D). To explore the potential roles of Nrf2 in tumor growth, we compared the tumor growth and survival of *Nrf2*^−/−^, WT, and Nrf2Tg mice injected subcutaneously with B16F10 melanoma, EL4 lymphoma, MC38 colon carcinoma, or TC-1 lung carcinoma cells. *Nrf2*^−/−^ mice displayed significantly decreased tumor growth (Figure 1B) and extended mouse survival (Figure S2E) compared with that in WT and Nrf2Tg mice. In addition, decreased tumor size and weight were observed in the *Nrf2*^−/−^ mice (Figure 1C). To test whether the enhanced anti-tumor responses in *Nrf2*^−/−^ mice were mediated by T cells, we depleted T cells using an α-CD3 antibody (Figure S2F) and found that T cell depletion significantly increased tumor growth in *Nrf2*^−/−^ mice (Figure 1D). In addition, T cell depletion did not prolong the survival of tumor-bearing *Nrf2*^−/−^ mice (Figure S2H). T cells, but not B cells (Figure S2G), played a crucial role in tumor rejection in *Nrf2*^−/−^ mice (Figure 1D). Thus, Nrf2 deletion promotes anti-tumor responses in a T cell-dependent manner. Next, we determined the cytokine profiles of WT and *Nrf2*^−/−^ TI T cells at the mRNA and protein levels and observed an increase in inflammatory cytokines (including *Ifng* and *Il-17*) and a decrease in anti-inflammatory cytokines (including *Il-4* and *Il-10*) in TI *Nrf2*^−/−^ T cells (Figure 1E). Specifically, compared with TI *Nrf2*^−/−^ T cells, TI WT and Nrf2Tg T cells exhibited significant reductions in the expression of inflammatory proteins, such as interferon γ (IFNγ) (Figures 1F and S2I). These results indicate that Nrf2 deficiency enhances the activity of TI T cells.

### Enhanced anti-tumor responses of *Nrf2*^−/−^ CD8^+^ T cells

**Figure 2:**
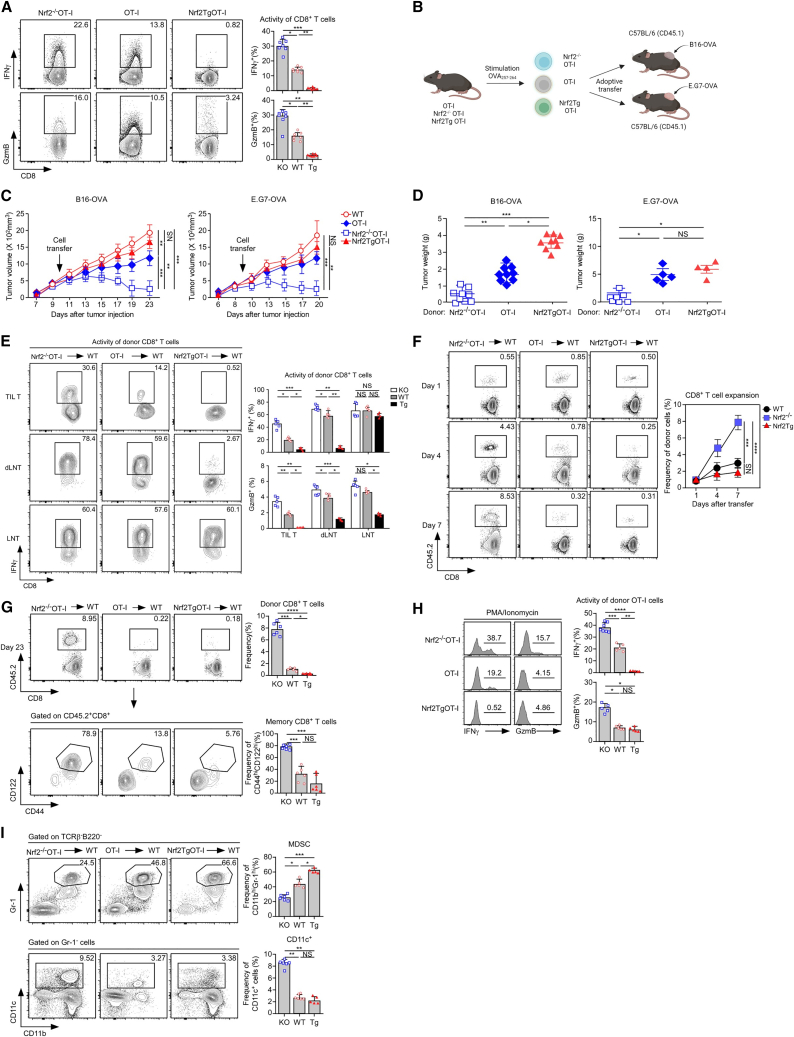
Nrf2-deficient CD8^+^ T cells display enhanced anti-tumor responses (A) Nrf2 effect on antigen-specific CD8^+^ T cell responses. CD8^+^ LN T cells from *Nrf2*^−/−^OT-I, OT-I, and Nrf2TgOT-I cells are stimulated for 16 h with OVA_257–264_ and assessed for IFNγ and GzmB expression by intracellular staining. IFNγ and GzmB profiles are representative of six independent experiments (left). The bar graph represents the percentage of IFNγ- or GzmB-producing OT-I cells (right, mean ± SEM). (B) Schematic of the experimental setup of *Nrf2*^−/−^OT-I, OT-I, and Nrf2TgOT-I generation and transfer to B16-OVA- or E.G7-OVA-bearing mice. (C and D) CD8^+^ LN T cells from *Nrf2*^−/−^OT-I, OT-I, and Nrf2TgOT-I cells are stimulated for 2 days with OVA_257–264_. Stimulated OT-I cells were adoptively transferred into tumor-bearing mice 10 days after subcutaneous (s.c.) challenge of B16-OVA (three independent experiments, *n* ≥ 4 mice/group) or E.G7-OVA (four independent experiments, *n* ≥ 4 mice/group). (C) Tumor volume is measured every 2–3 days. (D) Tumor weight is measured at the end of the experiment (mean ± SEM). (E) TIL T cells, dLN T, and LN T cells are isolated 23 days after B16-OVA challenge and stimulated with OVA_257–264_ for 16 h. IFNγ and GzmB expression are analyzed in donor OT-I cells using intracellular staining. Contour plots are representative of three independent experiments (*n* ≥ 4 mice/group, left). The bar graph represents the summary of three independent experiments (*n* ≥ 4 mice/group, means ± SEM). ∗*p* < 0.05, ∗∗*p* < 0.01, ∗∗∗*p* < 0.001. (F) Donor OT-I cells are traced in blood collected 1, 4, and 7 days after adoptive transfer. Contour plots are representative of three independent experiments (*n* ≥ 5 mice/group, left). Expansion kinetics of donor OT-I cells are shown by line graph, which is representative of three independent experiments (*n* = 5 mice/group, mean ± SEM). ∗*p* < 0.05, ∗∗*p* < 0.01, ∗∗∗*p* < 0.001, ∗∗∗∗*p* < 0.001. (G) Maintenance of donor OT-I cells in the spleen 23 days after B16-OVA challenge. Shown are CD8 versus CD45.2 profiles of CD4^−^TCRβ^+^-gated splenocytes (top) and CD44 versus CD122 profiles of donor OT-I cells (bottom). Contour plots are representative of two independent experiments (*n* ≥ 5 mice/group, left). The bar graph presents the summary of two independent experiments (*n* ≥ 5 mice/group, mean ± SEM, right). (H) Splenocytes isolated 23 days after B16-OVA challenge are stimulated with PMA/ionomycin, and IFNγ and GzmB expression was assessed in donor OT-I cells using intracellular staining. Histograms are representative of three independent experiments (*n* ≥ 5 mice/group, left). The bar graph presents the summary of three independent experiments (*n* ≥ 5 mice/group, mean ± SEM, right). (I) Percentages of Gr-1^hi^CD11b^hi^ MDSC subsets and the population of CD11c^+^ DCs in the spleen are analyzed gated on TCRβ^−^B220^−^ cells and gated on Gr-1^−^ cells, respectively. Contour plots are representative of two independent experiments (*n* ≥ 5 mice/group, left). The bar graph presents the summary of three independent experiments (*n* ≥ 5 mice/group, mean ± SEM, right). All data shown represent the summary of the error of the mean of independent experiments (∗*p* < 0.05, ∗∗*p* < 0.01, ∗∗∗*p* < 0.001, ∗∗∗∗*p* < 0.0001).

To gain further insight into the effects of Nrf2 in CD8^+^ T cells, we assessed the anti-tumor activity of antigen-specific CD8^+^ T cells using Nrf2-deficient or -overexpressing OT-I mice. *Nrf2*^−/−^OT-I, OT-I, and Nrf2TgOT-I cells were stimulated with an Ovalbumin (OVA_257–264_) antigen, and their cytokine profiles were examined *in vitro*. *Nrf2*^−/−^OT-I cells produced more IFNγ and GzmB than OT-I and Nrf2TgOT-I cells (Figure 2A). As Nrf2 deficiency improved cytokine production, we determined the *in vivo* anti-tumor activity of Nrf2-deficient OT-I cells using a solid tumor model with B16-OVA and E.G7-OVA (Figure 2B). The transfer of activated *Nrf2*^−/−^OT-I cells demonstrated superior anti-tumor effects compared with those of either activated OT-I or Nrf2TgOT-I cells (Figures 2C and 2D). The enhanced killing effects of *Nrf2*^−/−^OT-I cells were further confirmed by significantly reduced tumor weight (Figure 2D), and the total TI lymphocyte (TIL) number of *Nrf2*^−/−^OT-I cells significantly increased compared with OT-I or Nrf2TgOT-I cells (Figure S3A). Notably, *Nrf2*^−/−^OT-I effector cells highly expressed IFNγ and granzyme B (GzmB) compared with either OT-I or Nrf2TgOT-I cells in TILs and draining lymph nodes (dLNs) of B16-OVA-bearing (Figure 2E) or E.G7-OVA-bearing (Figure S3B) mice. We measured the frequency of the transferred effector OT-I cells in the blood and spleen at intervals after infusion. Overall, transferred OT-I cells were generally expanded in blood 1, 4, and 7 days after infusion into the B16-OVA-bearing (Figure 2F) and E.G7-OVA-bearing (Figure S3C) mice. *Nrf2*^−/−^OT-I effector cells were highly expanded and persisted over time compared with either OT-I or Nrf2TgOT-I cells, which were rarely detected 23 days after transfer to B16-OVA-bearing (Figure 2G) and E.G7-OVA-bearing (Figure S3D) mice. We evaluated the frequency of CD44- and CD122-expressing memory CD8^+^ T cells and found that the majority of long-lived *Nrf2*^−/−^OT-I cells had the CD44^hi^CD122^hi^ phenotype compared to the OT-I or Nrf2TgOT-I group in both tumor models (Figures 2G and S3D). We then analyzed the functional activity of the persisting OT-I cells with phorbol 12-myristate 13-acetate (PMA)/ionomycin and found that *Nrf2*^−/−^OT-I cells produced more IFNγ and GzmB than OT-I and Nrf2TgOT-I cells (Figures 2H and S3E). These data indicate that donor *Nrf2*^−/−^CD8^+^ T cells retained their functional properties. To assess the bystander effect of *Nrf2*^−/−^ T cells in these models, MDSCs or dendritic cells (DCs) in tumor-bearing mice were analyzed using flow cytometry. The frequency of Gr-1^hi^CD11b^hi^ MDSCs was significantly lower in the *Nrf2*^−/−^OT-I group than in the OT-I and Nrf2TgOT-I groups, whereas the frequency of CD11c^+^ DCs was dramatically increased in the *Nrf2*^−/−^OT-I group (Figures 2I and S3F). These data indicate that empowered *Nrf2*^−/−^ T cells induce an immune-favorable TME with regulation of DCs and MDSC differentiation, resulting in improved tumor rejection.

### Upregulation of ROS-sensing Nrf2 expression inhibited CD8^+^ T cell responses

**Figure 3:**
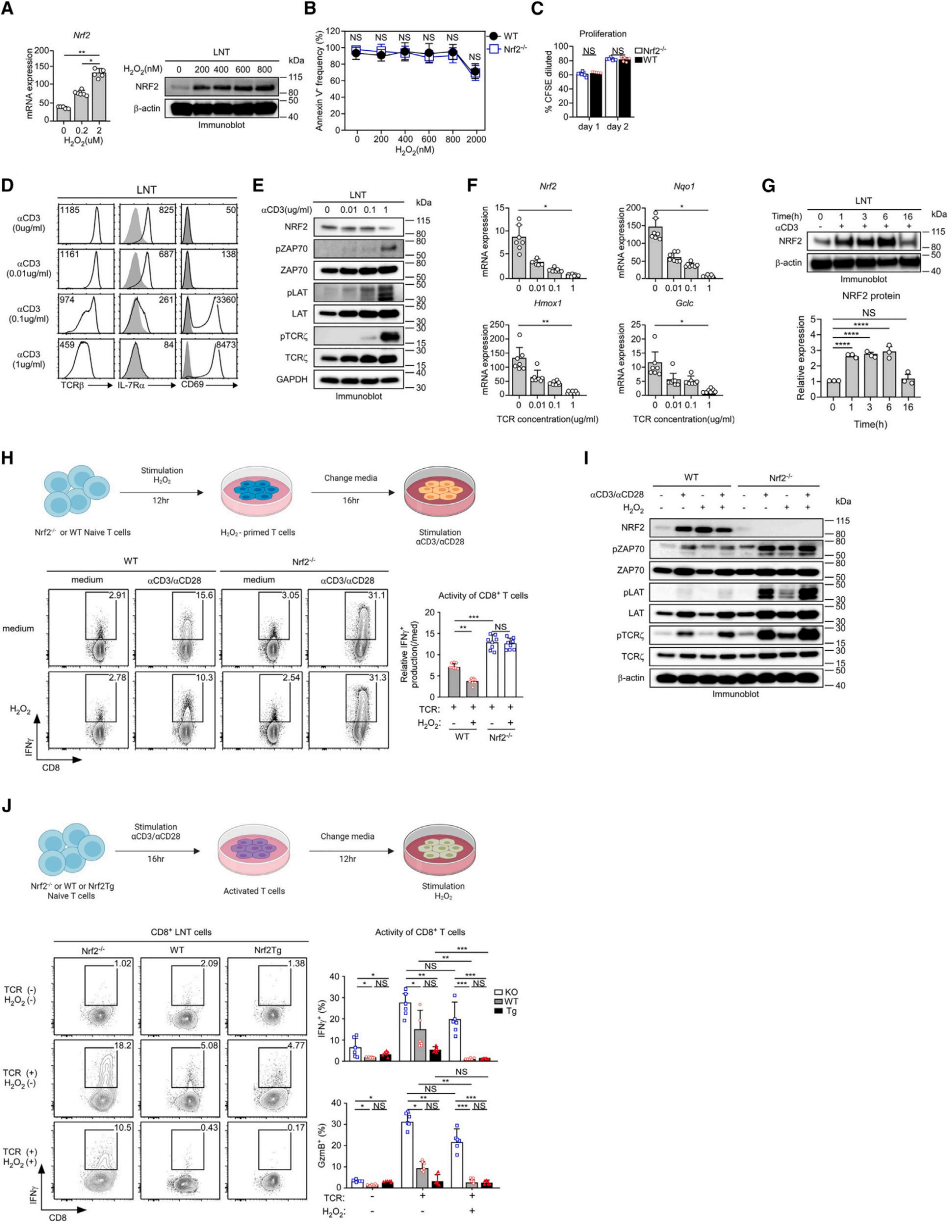
The ROS-Nrf2 axis regulates CD8^+^ T cell responses (A) *Nrf2* is induced by ROS in a dose-dependent manner. After WT CD8^+^ T cells are stimulated with H_2_O_2_ for 16 h, Nrf2 mRNA (left) and protein (right) levels are analyzed by RT-qPCR and western blot assay, respectively. The results summarize four individual experiments. (B) WT and *Nrf2*^−/−^ LN T cells are incubated with the indicated concentration of H_2_O_2_ for 16 h. The cell viability is determined using Annexin V staining. The graph shows a summary of three independent experiments. (C) CFSE-labeled WT and *Nrf2*^−/−^ LN T cells are stimulated with α-CD3/α-CD28. CFSE dilution is analyzed at the indicated time points. The results summarize three independent experiments. (D) LN T cells are stimulated with the indicated concentration of α-CD3 for 16 h. Surface expression of TCRβ, IL-7Rα, and CD69 in activated T cells is determined using FACS staining. Histograms are representative of three independent experiments. (E) NRF2 expression and TCR signaling pathways in differentially activated T cells are analyzed by immunoblotting. The blot is representative of five independent experiments. GAPDH is used as the loading control. (F) The expression of *Nrf2* and its target genes in differentially activated CD8^+^ T cells is evaluated using RT-qPCR. Data show the means ± SEM of five independent experiments (∗*p* < 0.05, ∗∗*p* < 0.01). (G) LN T cells are stimulated with α-CD3 (0.1 μg/mL) for the indicated times, and the kinetics of *NRF2* expression are analyzed by immunoblotting (top). β-Actin is used as the loading control. The blot is representative of three independent experiments. The bar graph presents the summary of three independent experiments (mean ± SEM, bottom, ∗∗∗∗*p* < 0.0001). The expression of NRF2 is normalized to that of β-actin. (H) Experimental scheme of the H_2_O_2_-primed T cell activity (top). WT and *Nrf2*^−/−^ naive T cells are incubated with 600 nM H_2_O_2_ for 12 h, and then H_2_O_2_-primed T cells are stimulated with α-CD3/α-CD28 (0.1 μg/mL) for 16 h. Intracellular IFNγ is analyzed (bottom). The bar graph presents the summary of six independent experiments (relative IFNγ production = TCR(+)H_2_O_2_(−) or TCR(+)H_2_O_2_(+)/TCR(−)H_2_O_2_(−), mean ± SEM, right). (I) TCR signaling in H_2_O_2_-primed T cells upon TCR stimulation. Immunoblot analysis of total and phosphorylated Zap70, LAT, and TCRζ in WT and *Nrf2*^−/−^ T cells stimulated under the indicated condition. β-Actin is used as the loading control. The blot is representative of four independent experiments. (J) Experimental scheme of the activated T cell activity under H_2_O_2_ treatment (top). Naive T cells from WT, *Nrf2*^−/−^, and Nrf2Tg mice are stimulated with α-CD3/α-CD28 (0.1 μg/mL) for 16 h, and then activated T cells are incubated with 600 nM H_2_O_2_ for 12 h and assessed for IFNγ expression by intracellular staining (bottom). The bar graph of IFNγ and GzmB production summarizes five independent experiments (right, mean ± SEM). ∗*p* < 0.05, ∗∗*p* < 0.01, ∗∗∗*p* < 0.001.

As in a previous study,^8^ we first confirmed that splenic MDSCs from tumor-bearing mice secreted more H_2_O_2_ than MDSCs from tumor-free mice (Figure S4A). The expression of H_2_O_2_ was comparable between *Nrf2*^−/−^ and WT MDSCs (Figure S4B). H_2_O_2_ increased Nrf2 mRNA and protein expression in CD8^+^ T cells (Figure 3A); however, survival analysis showed that Nrf2 deficiency had no significant effect on cell apoptosis (Figure 3B). In addition, we assessed the effect of Nrf2 on T cell proliferation using a carboxyfluorescein diacetate succinimidyl ester (CFSE) dilution assay and found that WT and *Nrf2*^−/−^ CD8^+^ T cells were comparably proliferated upon TCR stimulation (Figure 3C). To gain further insight into the relationship between Nrf2 levels and T cell activation, we examined the expression level of Nrf2 under different T cell activity by adjusting the strength of TCR signaling. Consequently, CD69 expression increased, whereas TCRβ and interleukin-7 receptor α (IL-7Rα) expression gradually decreased with increasing α-CD3 concentration (Figure 3D). Nrf2 expression was significantly downregulated in hyperactivated T cells with the highest levels of phosphorylated ZAP70, LAT, and TCRζ (Figure 3E). Concordantly, expression of *Nrf2* and its targets *Nqo1*, *Hmox1*, and *Gclc*^15^ was inversely downregulated by TCR strength (Figure 3F). We examined and compared the kinetics of Nrf2 expression in CD8^+^ T cells following TCR stimulation to clarify the regulatory function of Nrf2 in the context of complex cellular immune responses. Nrf2 expression increased in a time-dependent manner after CD8^+^ T cell priming until 6 h, whereas it decreased at 16 h (Figure 3G). Subsequently, we sought to determine the mechanism underlying the role of Nrf2 in T cell responses. Whereas IFNγ production in H_2_O_2_-primed WT CD8^+^ T cells was significantly suppressed even upon subsequent TCR stimulation, H_2_O_2_-primed *Nrf2*^−/−^ CD8^+^ T cells produced amounts of IFNγ comparable to medium-primed *Nrf2*^−/−^ CD8^+^ T cells (Figure 3H). H_2_O_2_-mediated Nrf2 induction in WT CD8^+^ T cells suppressed TCR signaling pathways with decreased induction of phosphorylated ZAP70, LAT, and TCRζ. Conversely, TCR signaling molecules in *Nrf2*^−/−^ CD8^+^ T cells were highly activated even in H_2_O_2_ conditions (Figure 3I). Activated *Nrf2*^−/−^ CD8^+^ T cells maintained the potential productivity of IFNγ (Figure 3J) and GzmB (Figure S4C) even in H_2_O_2_-rich conditions similar to the TME, while WT and Nrf2Tg CD8^+^ T cells showed reduced productivity. These findings suggest that ROS-sensing Nrf2 plays a role in inhibiting TCR signaling and that Nrf2-deficient CD8^+^ T cells sustain their effector functions and maintain normal activation even in ROS-rich conditions.

### Bioinformatic analysis of TI *Nrf2*^−/−^ effector T cells

**Figure 4:**
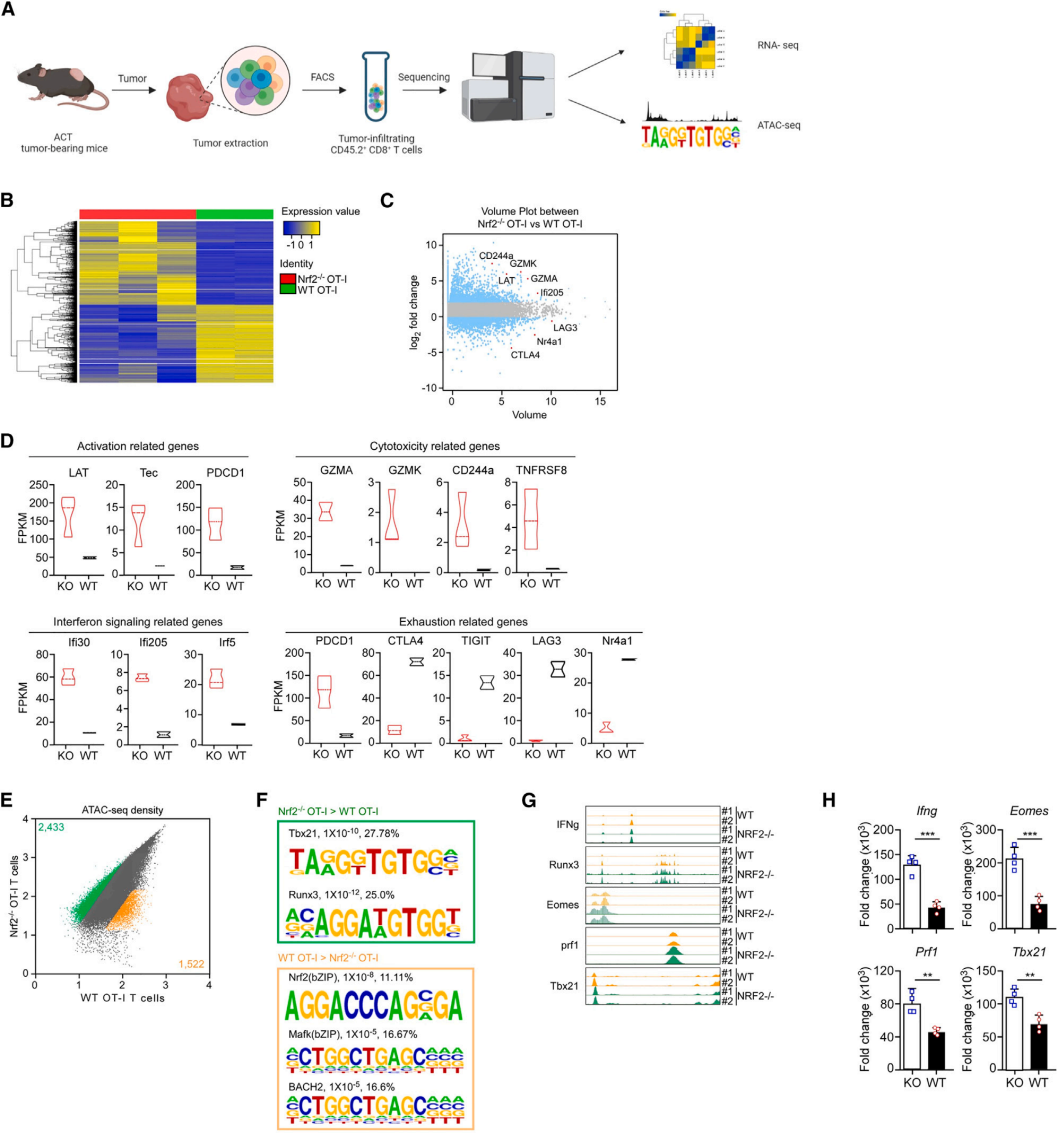
Gene expression and chromatin accessibility profiles in TI *Nrf2*^−/−^OT-I cells (A) Schematic of the experimental setup of TI CD8^+^ T cell generation and bioinformatics analysis. (B) Heatmap of genes with opposing expression changes between *Nrf2*^−/−^OT-I and WTOT-I T cells. (C) Volume plots of genes differentially expressed in *Nrf2*^−/−^OT-I versus WTOT-I T cells. Differentially expressed genes (adjusted *p* < 0.05, fold change [log_2_ scale] ≥ 1 or ≤ −1) are highlighted; selected genes are labeled. Fold change values (log_2_ scale) of genes differentially expressed in Nrf2^−/−^OT-I T cells relative to WTOT-I T cells are compared to those of the corresponding values in cells ectopically expressing Nrf2. (D) Fragments per kilobase of exon model per million mapped fragments of activation-, cytotoxicity-, IFN-, and exhaustion-related genes in different groups. (E and F) Scatterplot of pairwise comparison of ATAC-seq density (Tn5 insertions per kilobase) between *Nrf2*^−/−^OT-I and WTOT-I T cells showing differentially accessible regions and associated *de novo* identified motifs. (G) Genome browser view of the hyperfunction locus of CD8^+^ T cells in all previously mentioned ATAC-seq samples. (H) RT-qPCR analysis of effector function-related genes of CD8^+^ T cells in *Nrf2*^−/−^OT-I and WTOT-I T cells (*n* = 4 mice/group). The expression of the target genes is normalized to that of *Rpl13*. Data show the mean ± SEM of two independent experiments (∗∗*p* < 0.01, ∗∗∗*p* < 0.001).

To investigate how Nrf2 functions as a TF to mediate transcriptional profiles of T cell activity in the TME, we performed RNA sequencing (RNA-seq) analysis on TI WTOT-I vs. TI *Nrf2*^−/−^OT-I cells (Figure 4A). The gene expression of *Nrf2*^−/−^OT-I cells significantly differed from that in WT cells (Figure 4B). These samples were distinguished by volume plots and hierarchical clustering analysis of their expression profiles (Figures 4B and 4C). Expression of activation-related genes (*LAT*, *Tec*, and *PDCD1*), cytotoxicity-related genes (*GZMA*, *GZMK*, *CD244a*, and *TNFRSF8*), and IFN signaling-related genes (*Ifi30*, *Ifi205*, and *Irf5*) was higher in *Nrf2*^−/−^OT-I cells than in WTOT-I cells, whereas the expression of exhaustion-related genes (*CTLA4*, *TIGIT*, *LAG3*, and *Nr4a1*) was substantially lower in Nrf2^−/−^OT-I cells than in WTOT-I cells (Figure 4D). Gene Ontology analysis indicated that Nrf2 deficiency enhanced the expression of genes associated with immune responses (Figure S5A) or T cell activation-associated pathways (Figure S5B), consistent with our observation that *Nrf2*^−/−^ CD8^+^ T cells improved tumor lysis and cytokine productivity. To further confirm the unique chromatin landscape associated with effector T cells and how it may be altered by Nrf2 deficiency, the assay for transposase-accessible chromatin using sequencing (ATAC-seq) was performed with WT and *Nrf2*^−/−^OT-I cells sorted from TILs. Concordantly, a substantial fraction of the regions (11.11%) with lower accessibility in TI *Nrf2*^−/−^OT-I cells contained Nrf2-binding motifs. A smaller subset contained BTB domain and CNC homolog 2 (BACH2)-binding sites, suggesting that Nrf2 maintains the accessibility of “naive-related” regions that bind BACH2 (Figures 4E and 4F). Regions more accessible to *Nrf2*^−/−^OT-I cells than to WTOT-I cells were enriched for consensus Tbx21-binding (27.78%) and Runx3-binding (25%) motifs (Figure 4F). Collectively, TI *Nrf2*^−/−^OT-I cells exhibited potent effector function, enhanced cytokine production, and enrichment of accessible chromatin for the binding motifs involved in effector function. Nrf2 deficiency was associated with the increased accessibility of an enhancer of the effector function-related genes (*Ifng*, *Runx3*, *Eomes*, *Prf1*, and *Tbx21*) transcription sites (Figure 4G). Consistent with the ATAC-seq peak, we observed that effector function-related gene expression was upregulated in TI *Nrf2*^−/−^OT-I cells (Figure 4H). The ATAC-seq peak marking this enhancer was diminished in TI WTOT-I cells compared with TI *Nrf2*^−/−^OT-I cells (Figure 4G). Collectively, these data indicate that Nrf2 could downregulate effector genes and upregulate naive-associated genes by direct or indirect regulation, resulting in inducing exhaustion of CTLs in the ROS-rich TME.

### Anti-tumor responses of Nrf2KD CAR-T cells in a human tumor xenograft model

**Figure 5:**
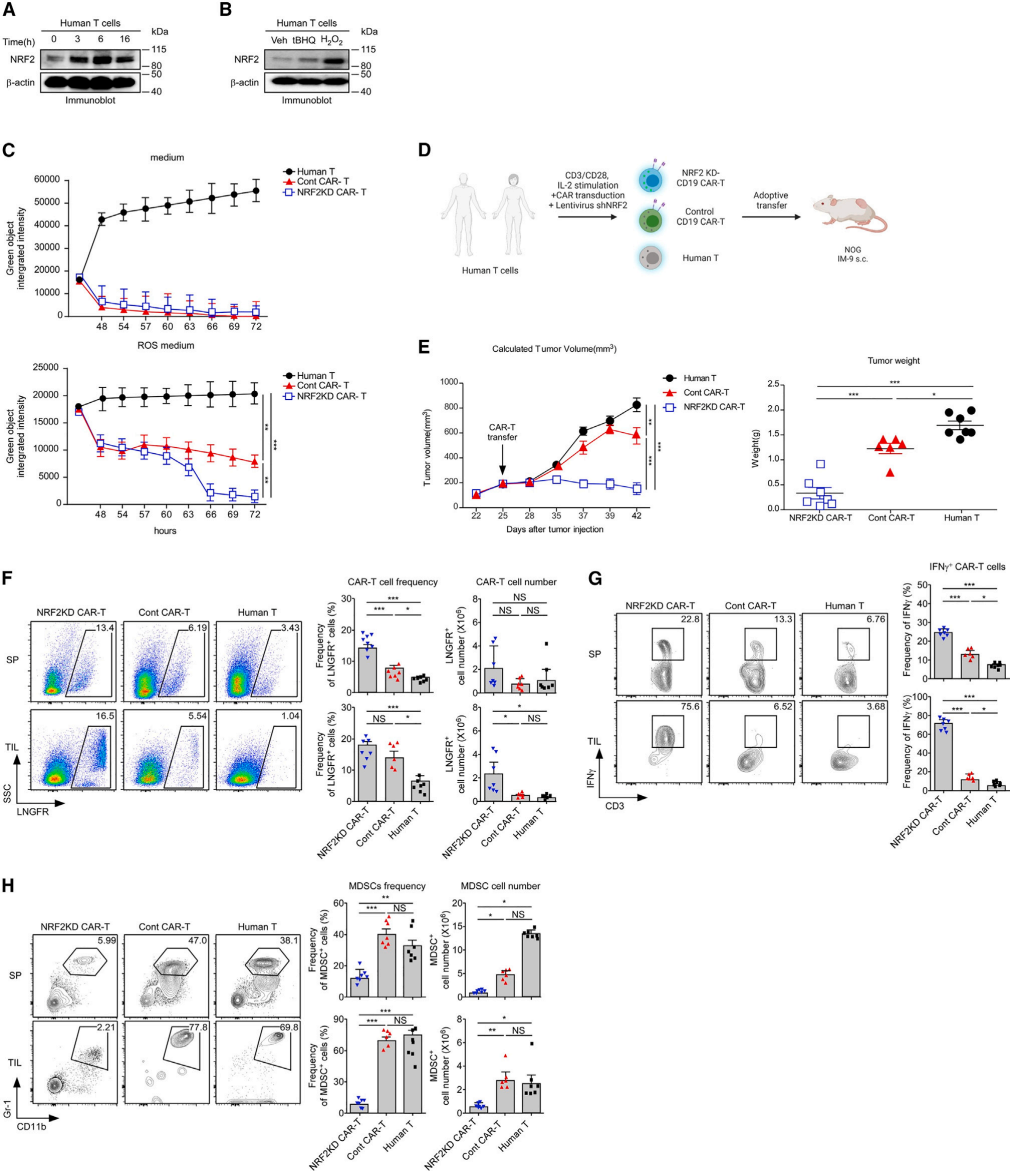
Nrf2 KD enhances *in vivo* efficacy of CAR-T cells against solid tumors (A and B) Human T cells from PBMC are stimulated with α-CD3/α-CD28 (1 μg/mL) for indicated times (A). Human T cells are incubated in 20 μM *tert*-Butylhydroquinone (tBHQ) and 100 μM H_2_O_2_ medium for 16 h (B). Cultured T cells are harvested and assessed for human Nrf2 by immunoblotting. β-Actin is used as the loading control. The blot is representative of three independent experiments. (C) Cytotoxicity assay using Nrf2KD-CAR-T cells and IM-9-zsgreen cells cocultured at a 1:1 ratio in medium (top) and 20 μM H_2_O_2_ (bottom). Each data point represents a mean of triplicate samples, and error bars represent SEM. The results are representative of three independent experiments. (D) Schematic of the CAR-T cell transfer experiments. (E) Human T, control CAR-T, and Nrf2KD-CAR-T cells are transferred to IM-9-zsgreen tumor-bearing NOG mice, and tumor growth is monitored twice a week. Tumor weight is measured at the end of monitoring (day 42). The results are a summary of four independent experiments (*n* = 7 mice/group). Data are represented as the mean ± SEM of four independent experiments. (F) LNGFR^+^ CAR-T cells are traced in the spleen and TILs 42 days after tumor injection. Dot plots are representative of three independent experiments (*n* = 7 mice/group). The bar graph summarizes LNGFR^+^ CAR-T cell frequency and numbers in the spleen and TILs. Data are representative of four independent experiments. (G) Splenocytes and TILs isolated from the indicated groups are stimulated with PMA/ionomycin, and IFNγ expression was assessed by intracellular staining. Contour plots are representative of four independent experiments (*n* = 7 mice/group). The bar graph shows the percentage of IFNγ-producing CAR-T cells (right, mean ± SEM). (H) MDSCs (Gr-1^hi^CD11b^hi^) from the spleen and TILs of tumor-bearing NOG mice. Contour plots are representative of four independent experiments (*n* = 7 mice/group). The bar graph represents the percentage and numbers of MDSCs (right, mean ± SEM). ∗*p* < 0.05, ∗∗*p* < 0.01, ∗∗∗*p* < 0.001.

To translate the Nrf2 effect into human T cell immunotherapy, we assessed the kinetics of NRF2 expression in stimulated human CD8^+^ T cells. We confirmed that human NRF2 expression was upregulated upon ROS and TCR stimulation, similar to that in murine CD8^+^ T cells (Figures 5A and 5B). To find an optimal xenograft animal model with the MDSC TME, we investigated the frequency of MDSCs following solid tumor formation in immunodeficient animal models, including NSG and NOG mice (Figure S6A), and found that MDSCs were significantly infiltrated or differentiated into the TME of NOG mice (Figure S6A). ROS production was significantly induced in the splenic MDSC from tumor-bearing NOG mice compared to NSG mice (Figure S6B). To confirm whether the anti-tumor efficacy of CAR-T cells is suppressed by MDSCs, CAR-T cells were transferred to tumor-bearing NOG and NSG mice. Anti-tumor responses of CAR-T cells were remarkably reduced in NOG mice in which MDSCs were generated, but not in NSG mice (Figure S6C). This implies that cytotoxic effects of CAR-T cells are critically influenced by the ROS-rich TME through MDSCs.

To determine whether Nrf2 targeting CAR-T cells could overcome the ROS-rich immunosuppressive TME formed by MDSCs, we generated Nrf2KD human CD19-CAR-T (Nrf2KD CAR-T) cells using the short hairpin RNA (shRNA)-Nrf2-CD19 CAR vector (Figures S6D–S6F). We evaluated the *in vitro* cytotoxicity of Nrf2KD CAR-T cells and confirmed that Nrf2KD CAR-T cells were effective against IM-9 regardless of H_2_O_2_ treatment, whereas WT CAR-T cells significantly lost cytotoxicity after H_2_O_2_ treatment (Figure 5C). After *in vivo* CAR-T cell transfer to an IM-9 xenograft NOG model (Figure 5D), Nrf2KD CAR-T cells exhibited substantially higher anti-tumor efficacy than control CAR-T and human T cells (Figures 5E and S7A). Consistent with the murine tumor model, numbers of Nrf2KD CAR-T cells significantly increased in the spleen and TILs compared control CAR-T cells, which were rarely detected after transfer (Figure 5F). Moreover, IFNγ production by Nrf2KD CAR-T cells was substantially enhanced in the spleen and TILs (Figure 5G). Consistent with fluorescence-activated cell sorting (FACS) data, serum IFNγ levels were also increased in the Nrf2KD CAR-T cell group compared with the other groups (Figure S7B), while the levels of IL-6 and IL-1β were comparable across all groups (Figure S7C). Notably, the frequency and number of MDSCs in the spleen and TILs of the Nrf2KD CAR-T cell group were significantly lower than those in the control CAR-T and human T cell groups (Figure 5H). ROS productivity was significantly reduced in the Nrf2KD CAR-T cell group (Figure S7D). These data indicated that targeting Nrf2 in CAR-T cell immunotherapy can promote the therapeutic effect on solid cancers.

## Discussion

Despite reports on the suppression of T cell functions within the ROS-rich TME, comprehensive mechanistic insights into the inhibitory mechanisms have not been fully understood. In this study, we demonstrated the inhibitory roles and molecular evidence of Nrf2 as a regulator of CD8^+^ T cell responses in the TME. Our findings confirmed that Nrf2 proteins, which are established regulators of antioxidant responses, also participate in the inhibition of CTL functions. Therefore, the novel regulator of the CD8^+^ T cell response identified in this study can serve as a possible target to overcome the limitations of applying anti-tumor ACT to patients with solid tumors.^30^^,^^31^

CTLs induce potent anti-tumor immune responses with the capability to eradicate tumors.^32^^,^^33^ Thus, studies on CD8^+^ T cell immunity within tumor environments is closely tied to understanding their activation, proliferation, and survival within the TME.^34^ The TME limits functional T cell responses via diverse immunosuppressive and physical barriers.^34^^,^^35^^,^^36^^,^^37^^,^^38^ Therefore, we hypothesized that the ROS-rich TME not only affects tumors but also TI T cells. Heterogeneous cell populations, including TAMs, MDSCs, and tumor cells within a solid tumor mass generate a ROS-rich milieu that attenuates the anti-tumor function of TI T cells.^8^^,^^9^^,^^39^ Therefore, ROS-responsible factors in T cells could be potential candidates influencing the effector functions of T cells. The role of ROS in the regulation of T cell responses has been extensively reported for T cell hyporesponsiveness, apoptosis, and activation.^13^^,^^30^^,^^40^^,^^41^^,^^42^ Nrf2 plays a role in cell protection by inducing antioxidant-related genes in response to OS, such as ROS.^43^^,^^44^ Interestingly, Nrf2 also plays a role in T cell differentiation and responses.^45^^,^^46^ Notably, single-cell RNA-seq (scRNA-seq) data from patients with melanoma have shown a preferential expression of Nrf2 in highly exhausted TI CD8^+^ T cells compared to less exhausted cells^26^ as well as a potential expression of signature immune checkpoint genes.^26^^,^^47^^,^^48^^,^^49^ Based on these studies, we hypothesized that a ROS-rich TME might influence Nrf2 expression and activation in TI CD8^+^ T cells, potentially leading to the disruption of cytotoxic effector functions. To confirm this hypothesis, we initially investigated Nrf2 expression in the immune cells within a murine solid tumor model and found a significant and specific upregulation of Nrf2 in TI T cells, in line with previous scRNA-seq data from cancer patients.^26^

The accumulation of ROS in the TME can be induced,^8^^,^^9^^,^^12^ resulting in potent manifestation of immunosuppressive and tumorigenic activities.^50^^,^^51^ These outcomes include various functions: (1) deprivation of essential amino acids for T cell proliferation and anti-tumor responses, (2) nitration of TCR and chemokines necessary for T cell trafficking and infiltration into tumor sites, (3) a reduction in the expression of the TCR-ζ chain, consequently inhibiting TCR-antigen recognition, and (4) upregulated expression of immunosuppressive cytokines such as transforming growth factor β (TGF-β) and IL-10.^52^^,^^53^^,^^54^^,^^55^ Thus, ROS emerge as key negative regulators in the anti-tumor CTL response. To confirm whether the suppression of anti-tumor CTL function induced by ROS producing-MDSCs is mediated through Nrf2, it is imperative to initially determine the distribution of MDSCs within the tumor mass and whether they serve as the source of ROS. Consistent with a previous study,^8^ we corroborated that ROS were produced by TI MDSCs and demonstrated that ROS levels remained relatively consistent irrespective of the presence or absence of Nrf2 expression. Although the detrimental effect of ROS on T cell activation is established,^13^^,^^14^ the intricate molecular mechanisms dictating T cell responses to ROS have not been fully revealed. Nrf2, recognized for its pivotal role in orchestrating an antioxidant response against ROS-induced OS,^56^^,^^57^ possibly extends this response to TI T cells exposed to ROS-rich environments, such as those found in solid tumors. Consistent with previous studies,^14^^,^^58^ we observed a significant augmentation of Nrf2 expression in T cells in response to ROS. Notably, our investigation unveiled a reciprocal relationship between T cell activation and Nrf2 expression, with Nrf2 expression diminishing as T cell activation increased. Notably, this observation suggests that the upregulation of Nrf2 expression in T cells within ROS-rich environments may contribute to the phenotypic transition of functional T cells into a dysfunctional state. Although activated WT T cells were functionally suppressed in OS media, activated *Nrf2*^−/−^ T cell function was maintained even under similar conditions, indicating that Nrf2 may function as a negative regulator of T cell effector function. Our findings align with the results of *in vivo* adoptive T cell transfer experiments in solid tumor-bearing mice. Notably, *Nrf2*^−/−^ TI CTLs or Nrf2KD-CAR-T cells exhibited superior effector functions compared to their exhausted WT CTLs or WT CAR-T cells. Moreover, the enhanced resistance of effector T cells to ROS, achieved by Nrf2 deletion, was evident in reduced tumor growth rates and improved survival outcomes. These findings collectively underscore the potential of targeting Nrf2 as a strategy to enhance T cell effector function in the challenging microenvironment characterized by OS.

To define the molecular mechanism of the OS-induced inhibition of T cell responses, we delved into key components of TCR signaling, including TCRζ, LAT, and ZAP70.^59^ Our investigation unveiled ROS-dependent downregulation of these molecules in WT T cells, contrasting with the sustained expression in *Nrf2*^−/−^ T cells even under ROS-rich conditions. Notably, enhanced TCR signaling with highly phosphorylated signaling molecules was observed in *Nrf2*^−/−^ T cells, leading to potentiated effector functions. Our findings suggest that the expression and phosphorylation of TCR signaling molecules are intricately regulated in a Nrf2-dependent manner under ROS-rich conditions. However, comprehensive studies are required to elucidate the precise mechanisms through which Nrf2 governs the expression and phosphorylation of these molecules. Improved TCR signaling in *Nrf2*^−/−^ T cells translated into an enhanced production of cytotoxic cytokines. Our ATAC-seq analysis further demonstrated that the accessibility of genes associated with T cell activation was increased in *Nrf2*^−/−^ T cells, whereas inhibition-related genes showed higher accessibility in WT TI T cells. Additionally, cytokine profiling analysis discerned distinct expression patterns of anti-tumor cytotoxic cytokines based on Nrf2 expression. Although our findings provide valuable insights into the role of Nrf2 in modulating T cell function, further investigations are imperative to unravel the precise action mechanisms through which Nrf2 influences T cell activation.

Given the potential significance of Nrf2 as a key target to enhance the efficacy of CAR-T cell immunotherapy, our investigation focused on exploring the distribution of TI MDSCs contributing to the composition of the ROS-rich TME in tumor-bearing NOG and NSG mice, commonly employed for xenotransplantation.^60^ Although the two strains are indistinguishable in terms of immunodeficiency and susceptibility to xenotransplantation,^60^ our data revealed distinct distributions of MDSCs within the tumor. Specifically, there was a notable increase in the NOG host compared to the NSG host. This discrepancy was attributed to variations in the anti-tumor efficacy between Nrf2KD- and WT-CAR-T cells in NOG and NSG hosts. In the NSG host, characterized by a TME with a low frequency of MDSCs, the anti-tumor efficacy of Nrf2KD-CAR-T cells was comparable to that of WT-CAR-T cells. Conversely, in the NOG host with an MDSC-enriched TME, the anti-tumor efficacy of Nrf2KD-CAR-T cells exhibited substantial enhancement. This observation aligns with earlier findings suggesting that the anti-tumor responses of TILs are hindered by MDSCs,^8^^,^^61^ a phenomenon crucial in the context of the ROS-rich TME.^13^ Furthermore, the substantial reduction in intratumoral MDSCs observed in hosts treated with Nrf2KD-CAR-T cells suggests that the activation of TI T cells has the potential to modulate the infiltration or differentiation of immunosuppressive cells within the TME. These findings underscore the notion that the efficacy and survival of activated TI T cells can be further augmented through strategic manipulation of the TME.

In recent studies elucidating the role of Nrf2 in anti-tumor CD8^+^ T cell responses, Nishida et al. reported that metformin-induced mitochondrial ROS (mtROS) induction could enhance anti-tumor immunity through Nrf2 expression in CD8^+^ T cells. However, despite suggesting an association between metformin-induced mtROS increase and Nrf2 in the context of tumor rejection, there is insufficient direct and clear *in vitro* and *in vivo* data to substantiate this claim. Moreover, it fails to provide conclusive results demonstrating the role of Nrf2 in the *in vivo* anti-tumor immune response through CD8^+^ T cells.^28^ Renken et al. reported that pretreatment of TILs with auranofin enhanced anti-tumor response and preserved their antitumoral activity even under H_2_O_2_-rich conditions, speculating an involvement of Nrf2. However, a direct correlation between Nrf2 expression in T cells and anti-tumor responses has not been fully elucidated.^29^ More recently, Gnanaprakasam et al. suggested that asparagine restriction enhances anti-tumor immune responses of CD8^+^ T cells in an Nrf2-dependent manner.^62^ Notably, their findings contradict ours, as T cells overexpressing Nrf2 in our study demonstrated impaired anti-tumor responses. Despite model differences, such as transfection system and antigen variance, explaining these incongruities remains challenging. Crucially, we established a T cell-specific overexpression animal model, demonstrating that Nrf2 overexpression dampens the anti-tumor response, whereas, conversely, *Nrf2*^−/−^ promotes T cell-driven anti-tumor responses across multiple *in vivo* models. *Nrf2*^−/−^ cells exhibited resistance to H_2_O_2_-mediated suppression of proximal T cell signaling, and they underwent transcriptional and epigenetic reprogramming, becoming more activated and/or effector like, consistent with our *in vivo* data. In a human xenograft model optimized for MDSC infiltration, CD19-directed CAR-T cells with Nrf2KD displayed enhanced tumor infiltration and control. In summary, our findings establish a direct correlation between Nrf2 and the anti-tumor immune response of CD8^+^ T cells in multiple *in vivo* models, contributing to the robustness of our conclusions. The discrepancy with other studies underscores the complexity of the interplay between Nrf2 and anti-tumor immunity, warranting further investigation of the specific mechanisms and context-dependent effects.

Our research unequivocally establishes the pivotal role of Nrf2 in the immune evasion tactics employed by tumors through the OS induction. This study sheds light on Nrf2’s regulatory role on the anti-tumor immune response orchestrated by CD8^+^ T cells. Notably, we underscored the significance of conferring resistance to OS as a means of preserving the effector properties of tumor-specific T cells. Furthermore, we emphasize that the engineering of tumor-specific T cells equipped with OS resistance, as demonstrated through Nrf2KD, may alone suffice to drive robust and effective anti-tumor responses in solid tumor models. Although additional investigations employing actual solid tumor cell lines are warranted, our present findings hold the remarkable implication that the modulation of Nrf2 expression alone can trigger a sufficiently potent anti-tumor immune response against solid tumors. This discovery carries profound implications for the advancement of immunotherapeutic strategies. We firmly believe that this study contributes vital insights for enhancing existing therapeutic modalities and fostering the development of innovative approaches to combat solid tumors.

## Materials and methods

### Animals

C57BL/6 (B6) mice were obtained from Orient Bio, NOG (NOD.Cg-Prkdc^scid^ Il2rg^tm1Sug^/ShiJic) mice were obtained from Koatech, and OT-I, B6.SJL (CD45.1), and NSG (NOD.Cg-Prkdc^scid^ Il2rg^tm1Wjl^/SzJ) mice were obtained from The Jackson Laboratory. B6 *Nrf2*^−/−^ mice^63^ were provided by Dr. Joo (Pusan National University). Nrf2 Tg constructs were generated by ligating murine Nrf2 cDNA into hCD2 enhancer promoter-based vectors and inoculating fertilized B6 oocytes to generate Nrf2 Tg mice. The Nrf2Tg and *Nrf2*^−/−^ mice were bred with OT-I mice to generate OVA_257–264_-specific Nrf2-overexpressing or deficient CD8^+^ T cells. All experimental mice were maintained in a specific pathogen-free facility at Pusan National University (PNU) and were 6–8 weeks old and of either sex. All animal experiments were performed according to protocols approved by the PNU Institutional Animal Care and Use Committee (PNU-2019-2180, PNU-2020-2580, PNU-2021-2975, and PNU-2022-3183).

### Tumor cells and tumor growth

EL4 lymphoma, B16F10 melanoma, MC38 colon carcinoma, TC-1 lung carcinoma, B16-OVA, E.G7-OVA, and IM-9 cells were maintained as described by the ATCC. The mice were inoculated subcutaneously into the right back with 1 × 10^6^ tumor cells. To deplete T and B cells in tumor-bearing mice, mice were treated intraperitoneally with either α-CD3 (145-2C11) or α-B220 (RA3.3A1/6.1) or isotype control immunoglobulin G (IgG) once every 5 days. Tumor size was monitored twice a week, and tumor volume was calculated as follows: volume (mm^3^) = (3.14 × length × width × height)/6. The survival time was recorded when the mice became moribund and were euthanized.

### TIL isolation

Tumors were resected from the tumor-bearing mice, minced with scissors, and placed in a flask containing a dissociation solution of collagenase IV, hyaluronidase, and DNase IV (Sigma-Aldrich). The tumor mass within the flask was rotated, and red blood cells were removed using RBC lysis buffer. Cells were subjected to Histopaque (Sigma-Aldrich; density, 1.083 g/mL) density gradient centrifugation (Eppendorf Centrifuges, 1,025 × *g* for 20 min at 20°C). Lymphocytes were collected at the interface for further analysis or isolation.

### ROS detection

ROS were measured by detecting H_2_O_2_ using the Amplex Red Hydrogen Peroxide Assay Kit (Invitrogen), as described previously.^23^ Briefly, MDSCs were electronically sorted by gating on TCRβ^−^CD45R^−^CD11b^+^Gr1^+^ splenocytes or TILs from tumor-bearing or WT mice and incubated with PMA (Merck Millipore, 12.5 ng/mL) and 50 μL Amplex Red reagent. Plates were incubated at 37°C, and fluorescence (excitation at 530 nm and emission at 590 nm) was measured using a microplate reader (Tecan). A standard curve was generated using serial dilutions of 20 μM H_2_O_2_.

### Quantitative Real-Time PCR

T cells, B cells, and non-T/B (DN) cells from TILs were electronically sorted by gating on TCRβ^+^CD45R^−^ cells and TCRβ^−^CD45R^+^ and TCRβ^−^CD45R^−^ cells, respectively, using an FACSAria I or FACSAria III (BD Biosciences). T cells from dLNs and non-dLNs in tumor-bearing mice and WT LN T cells were electronically sorted by gating on TCRβ^+^CD45R^−^ cells. For analysis of mRNA levels from activated CD8^+^ T cells, naive CD8^+^ T cells isolated by BioMag goat α-mouse IgG beads and α-rat IgG beads (QIAGEN, Hilden, Germany) were stimulated with plate-bound α-CD3 (BioLegend; 0, 0.01, 0.1, and 1 μg/mL) or with H_2_O_2_ (0, 0.2, and 2 μM) for 16 h. Total RNA was isolated immediately using Ribospin (GeneAll). RNA was reverse transcribed into cDNA using oligo(dT) priming with a reverse transcription kit (GeneAll). RT-qPCR was performed using SYBR Green Master Mix (Bio-Rad) on a Light Cycler 96 real-time PCR system (Roche). Primer sequences were as follows: *Nrf1* (F: 5′-GACAAGATCATCAACCTGCCTGTAG-3′; R: 5′-GCTCACTTCCTCCGGTCCTTTG-3′), *Nrf2* (F: 5′-TAGATGACCATGAGTCGCTTGC-3′; R: 5′-TCAGCCAGCTGCTTGTTTTC-3′), *Nrf3* (F: 5′-GCAGGAGGAAAACGAGGAA-3′; R: 5′- GACCAATGTAGATGGCTCTCG-3′) *Nqo1* (F: 5′-GCATTGGCCACAATCCACCAG3′; R:5′-ATGGCCCACAGAGAGGCCAAA-3′), *Hmox1* (F: 5′-CACGCCAGCCACACAGCACTA-3′; R: 5′-GGCTGTTCGGGAAGG-3′), *Gclc* (F: 5′-GCACGGCATCCTCCAGTTCCT-3′; R: 5′TCGGATGGTTGGGGTTTGTCC-3′), *IFNγ* (F: 5′-TGGCTCTGCAGATTTTCATG; R: 5′-TCAAGTGGCATAGATGTGGAAGAA-3′), *IL-17a* (F: 5′-CTCCAGAAGGCCCTCAGACTAC-3′; R: 5′-GGGTCTTCATTGCGGTGG-3′), *IL-4* (F: 5′-CGAGGTCACAGGAGAAGGGA-3′; R: 5′-AAGCCCTACAGACGAGCTCACT-3′), *IL-10* (F: 5′-GGTTGCCAAGCCTTATCGGA-3′; R: 5′-ACCTGCTCCACTGCCTTGCT-3′), *Rpl13* (F: 5′-CGAGGCATGCTGCCCCACAA-3′; R: 5′-AGCAGGGACCACCATCCGCT-3′). The gene expression values were normalized to that of *Rpl13* in the same sample.

### Immunoblotting

LN T cells were stimulated with H_2_O_2_ or medium with or without plate-bound αCD3 for 16 h and lysed. T cells from human PBMCs were stimulated with αCD3 (1 μg/mL)/αCD28 (eBioscience, 1 μg/mL), tBHQ (Sigma-Aldrich, 20 μM), or H_2_O_2_ (Sigma-Aldrich, 100 μM). Cell lysates were resolved by SDS-PAGE on 12% acrylamide (Invitrogen) and then transferred to polyvinylidene fluoride (PVDF) membranes (Amersham Biosciences). The membranes were incubated with anti-NRF2 (D1Z9C), Zap70 (99F2), LAT (E3U6J), GAPDH (D16H11), phospho-Zap70 (Y319), phospho-LAT (Y191), and phospho-TCRζ (Y142) (1:1,000, Cell Signaling Technology) antibodies, followed by horseradish peroxidase (HRP)-conjugated anti-rabbit or anti-mouse IgG (1:5,000, Cell Signaling Technology) and TCRζ (6Β10.2) HRP-conjugated anti-mouse β-actin antibodies (1:2,000, Santa Cruz Biotechnology). Then, the membranes were incubated with enhanced chemiluminescence reagents (Amersham Biosciences, Amersham, Buckinghamshire, UK) and exposed using the LAS-3000 imaging system (Fujifilm, Minato-ku, Tokyo, Japan), ChemiDoc Imaging System (Bio-Rad), and an Amersham Imager 680 (Amersham Biosciences).

### Proliferation and survival assay

LN T cells were stimulated with different concentrations of H_2_O_2_ for 16 h and stained with Annexin V according to the manufacturer’s instructions (Thermo Fisher Scientific, eBioscience). LN T cells were labeled with CFSE (Thermo Fisher Scientific, Invitrogen) and stimulated with αCD3/αCD28. Cell division was assessed by flow cytometry for CFSE dilution.

### *In vitro* cytotoxicity assay

Zsgreen-expressing IM-9 target cells (IM9-zsgreen) were cocultured with CAR-T cells at a 1:1 E:T ratio for 72 h in the presence or absence of 20 μM H_2_O_2_. GFP fluorescence intensity was detected every 3 h using high-content screening (Thermo Fisher Scientific). The total integrated GFP intensity per well was used as a quantitative measure of viable target cells. The total integrated GFP intensity values were normalized to the GFP intensity at the starting point.

### Flow cytometry analysis

The cells were harvested, stained, and analyzed using Attune NxT (Thermo Fisher Scientific), FACSCanto II, and FACSAria III (Becton Dickinson). Dead cells were excluded using forward light-scatter gating and propidium iodide staining. The data were analyzed using FlowJo v.10 (Tree Star). LNGFR was labeled with an allophycocyanin (APC)-conjugated anti-LNGFR antibody (clone ME20.4-1.H4, Miltenyi Biotec, Germany). Antibodies with the following specificities were used for staining: CD45.1 (Ly5.1), CD45.2 (Ly5.2), TCRβ (H57-597), IL-2Rβ (TM-β1), granzyme B (NGZB), CD45R (B220), IL-7Rα (A7R34), and isotype control antibodies (all from Thermo Fisher Scientific, eBioscience); CD4 (GK1.5 and RM4.5), CD69 (H1.2F3), and CD8α (53-6-7) (BD Biosciences); CD11b (M1/70) and CD44 (IM7) (TONBO Bioscience); and IFNγ (XMG1.2), CD11c (N418), Ly6G/Ly6C (Gr-1) (RB6-8C5), IL-17A (TC11-18H10.1), CD3 (OKT3), CD4 (OKT4), CD8 (SK1), CD19 (HIB19), and IFNγ (4S.B3; BioLegend). APC-conjugated H-2Kb tetramers loaded with OVA_257–264_ and unloaded controls were obtained from the NIH tetramer. An anti-mouse CD16/32 antibody (2.4G2; BioLegend) was used to block the Fc receptor. For intracellular cytokine staining, the cells were stimulated with PMA (12.5 ng/mL) and ionomycin (1 μM, Thermo Fisher Scientific) in the presence of brefeldin A (BioLegend) and subsequently fixed and permeabilized with intracellular fixation buffer (Thermo Fisher Scientific, eBioscience).

### Adoptive cell transfer *in vivo* models

Nrf2^−/−^OT-I, OT-I, and Nrf2TgOT-I cells were stimulated with 10 ng/mL OVA_257–264_ for 2 days. Activated OT-I cells were transferred intravenously into B6.SJL mice (5 × 10^6^/mouse) in which B16-OVA or E.G7-OVA cells were implanted subcutaneously (s.c.) by injection 10 days before. Tumor-bearing mice were not conditioned for the ACT by total body irradiation or IL-2 administration. Tumors were followed by caliper measurements a few times a week: volume (mm^3^) = (3.14 × length × width × height)/6.

### RNA-seq

To analyze the function of CD8^+^ T cells in the TME, we sorted TI CD8^+^ T cells from tumor-bearing mice. Bulk RNA-seq with TI CD8^+^ T cells was performed by Macrogen (Seoul, Korea). FASTQ data were mapped to the reference genome hg19 using HISAT2 v.2.1.0 software. The number of reads per annotated gene was computed from the mapped reads using featureCounts v.1.6.4 software. The R package limma with voom method v.3.38.3 was used to normalize all datasets and analyze differential expression between the groups. Pearson’s correlation matrices and hierarchical clustering plots of differentially expressed genes (false discovery rate <0.1) were generated using the Instant Clue software. A heatmap of the selected genes associated with T cell function was generated using GraphPad Prism 8. Each row represents the *Z* scores of the normalized gene expression values for the selected genes. RNA-seq data were deposited in the Gene Expression database (GSE229992).

### ATAC-seq analysis

ATAC-seq was performed as described previously.^64^ Briefly, TI CD8^+^ T cells from tumor-bearing mice were sorted by FACS using FACSariaIII (BD Biosciences). ATAC-seq was performed using ActiveMotif (ActiveMotif, Carlsbad, CA, USA). ATAC-seq peaks were detected using the DANPOS2 deregion function^65^ and visualized using the University of California, Santa Cruz genome browser. Peak height was normalized against the Gapdh locus. The gain and loss regions were defined by a height q value of <0.01 and log2 fold change of >2. Associated genes were selected based on the region from upstream 3 kb to downstream 10 kb of the promoter. Pathway enrichment analysis was performed using DAVID.^64^

### Plasmid construction

To construct the lentiviral transfer vector encoding the CD19-specific CAR, the anti-CD19 scFv (FMC63) was fused by overlapping PCR to the CD8α spacer and transmembrane domains, the 4-1BB (CD137) or CD28 costimulatory domains, and the CD3ζ signaling domain. shRNA expression cassettes were added upstream of the pLV-EF-1α-ΔLNGFR-P2A-CD19 CAR vector in the antisense direction under the RNA polymerase III promoter (mU6). For Nrf2 KD, shRNA-expressing modules, controlled by the mU6 promoters, were cloned in upstream of the EF-1α promoter (Figure S6D).

### Generation of human CAR-T cells

Human PBMCs were obtained from healthy adults using protocols approved by our institutional review board (KH2017-39). Lenti-X 293 T cells were incubated in poly-D-lysine-coated dishes for 3 days before transduction. After 24 h, cells were co-transfected with the lentiviral transfer plasmid, pMDG.1 encoding a vesicular stomatitis virus G protein envelope, pRSVRev encoding Rev, and pMDLg/pRRE encoding Gag/Pol using Lipofectamine 2000 according to the manufacturer’s instructions. Lentiviral supernatants were collected 40 h after transfection, and cell debris was removed by centrifugation and immediately used to transduce T cells. PBMCs were collected from the whole-blood samples of healthy donors using SepMate tubes (STEMCELL Technologies) in accordance with the manufacturer’s instructions. PBMCs were stimulated with a plate-bound anti-CD3 antibody (clone OKT3, Bio X Cell), a soluble anti-CD28 antibody (clone CD28.2, Bio X Cell), or recombinant human IL-2 (rhIL-2, BMI Korea). Two days after stimulation, the activated T cells were transduced with lentiviral supernatants and protamine sulfate. After 24 h, the lentiviral supernatants were removed, and the transduced T cells were expanded. The percentage of transduced T cells was evaluated based on CD271 (LNGFR) expression 4 days after transduction. LNGFR^+^ T cells were isolated using a human CD271 MicroBead kit (Miltenyi Biotec) according to the manufacturer’s instructions. CAR^+^ LNGFR^+^ T cells were maintained in complete T cell medium with rhIL-2.

### *In vivo* xenograft models

IM-9 was injected s.c. into the right flank of NOG and NSG mice. When the tumor reached an average volume of 200–250 mm^3^, 1.5 × 10^6^ CAR-T cells or human T cells were transferred to tumor-bearing NOG and NSG mice. To examine the *in vivo* efficacy of Nrf2KD CAR-T cells against solid tumors, 1 × 10^7^ IM-9 zsgreen was injected s.c. into the right flank of NOG mice. When the tumor reached an average volume of 250–300 mm^3^, the mice were randomized for even distribution between groups and were infused intravenously with 1.5 × 10^6^ WT CAR-T, Nrf2KD CAR-T, and control human T cells. The tumor volume was calculated using the following formula: volume (mm^3^) = width × width × length/2. Mice were euthanized when the longest tumor diameter exceeded 20 mm.

### Statistical analysis

Data are presented as the mean ± SEM. Statistical differences were analyzed using two-tailed Student’s t tests or one-way ANOVA. Statistical significance was set at ∗*p* < 0.05, ∗∗*p* < 0.01, ∗∗∗*p* < 0.001, and ∗∗∗∗*p* < 0.0001. All statistical analyses were performed using GraphPad Prism.

## Data and code availability

The data for this manuscript have been deposited as a SuperSeries into the accession code GEO: GSE229994. Within this SuperSeries, processed data for the RNA-seq are available under accession code GEO: GSE229992 and raw and processed data for ATAC-seq of intratumoral WTOT-I vs. *Nrf2*^−/−^OT-I T cells under accession code GEO: GSE229990. The remaining data are available within the article, supplemental information, or source data file. Source data are provided with this paper.

## Acknowledgments

We thank Dr. Joo at the PNU, Dr. Chang at The University of Michigan Medical School, and the members of the Hong lab for critical review of this manuscript. This work was supported by the Basic Science Research Program through the 10.13039/501100003725National Research Foundation of Korea (NRF) funded by the 10.13039/501100003621Ministry of Science, ICT, & Future Planning (2023R1A2C2002435).

## Author contributions

C.H. conceived and designed the study. Y.J., J.A.S., J.W.J., H.K., S.M.L., J.H.R., J.J., S.K., S.-K.I., D.C., B.H.L., Y.H.K., C.D.K., C.H.K., and C.H. performed experiments and analyzed data. Y.J., J.A.S., J.W.J., H.K., S.M.L., S.K., and C.H. analyzed and interpreted the results. Y.J. and C.H. wrote the manuscript. All authors read and approved the manuscript.

## Declaration of interests

C.H. received funding from NeoImmuneTech, Inc. D.C., S.-K.I., and B.H.L. are currently employed by NeoImmuneTech, Inc.

## Supplemental information

Document S1. Figures S1–S7 and Supplemental materials and methods

Document S2. Article plus supplemental information
