# Supplementary material for: Targeting ROS-sensing Nrf2 potentiates anti-tumor immunity of intratumoral CD8+ T and CAR-T cells
Source: Mol Ther. 2024 Aug 22;32(11):3879–94. doi: 10.1016/j.ymthe.2024.08.019 (PMC11573615; doi:10.1016/j.ymthe.2024.08.019)
Supplement: Document S1. Figures S1–S7 and Supplemental materials and methods [file mmc1.pdf]

YMTHE, Volume *32*

Supplemental Information

Targeting ROS-sensing Nrf2 potentiates anti-tumor immunity of intratumoral CD8^{+} T and CAR-T cells

Yuna Jo, Ju A. Shim, Jin Woo Jeong, Hyori Kim, So Min Lee, Juhee Jeong, Segi Kim, Sun-Kyoung Im, Donghoon Choi, Byung Ha Lee, Yun Hak Kim, Chi Dae Kim, Chan Hyuk Kim, and Changwan Hong

Supplemental Information

MATERIALS AND METHODS

ELISA for cytokine quantification

Serum cytokines were detected using enzyme-linked immunosorbent assay (ELISA). Serum samples were collected from tumor-bearing mice that were transferred with CAR-T cells. The cytokine levels were measured using the following ELISA kits: murine IL-6 (BD Biosciences), murine IL-1β (R&D Systems), and human IFNγ and TNFα (BioLegend), following the manufacturer's instructions.

<u>**Supplemental Figures**</u>

Figure S1

A

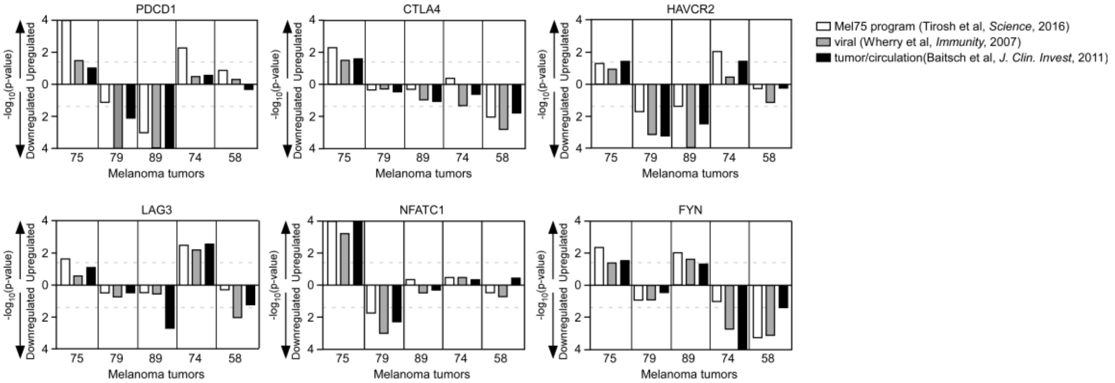

B

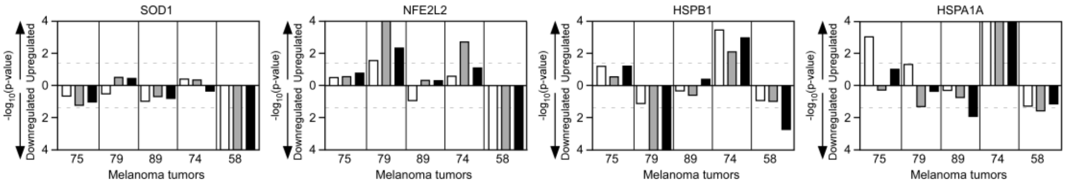

Figure S1. Tumor-specific activation-independent exhaustion program.

(A and **B)** Three values are shown for each tumor, corresponding to exhaustion scores based on the exhaustion gene sets derived from Mel75 analysis [1] , from Wherry et al. [2], and from Baitsch et al. [3] respectively. Tumor-specific associations with the exhaustion program, detected by co-expression across single cells, are not detected by the overall (bulk) tumor-specific expression in $CD8^+$ T cells. Genes with significant tumor-specific up- or down-regulation in high-exhaustion cells (FDR < 0.05 in each tumor, based on the median of the two exhaustion scores), were divided to three classes (bars) based on the differences in their overall expression level in $CD8^+$ T cells among the different tumors (White: Mel75 program (Tirosh et al. *Science*, 2016), Grey: Vial (Wherry et al. *Immunity*, 2007), Black: tumor/circulation (Baitsch et al., *J. Clin. Invest*, 2011)). Bar plots showing the significance of tumor-specific variation for high exhaustion program-related genes (**A**) and oxidative stress-related genes (**B**). Dashed lines indicate significance thresholds that correspond to P < 0.05.

Figure S2

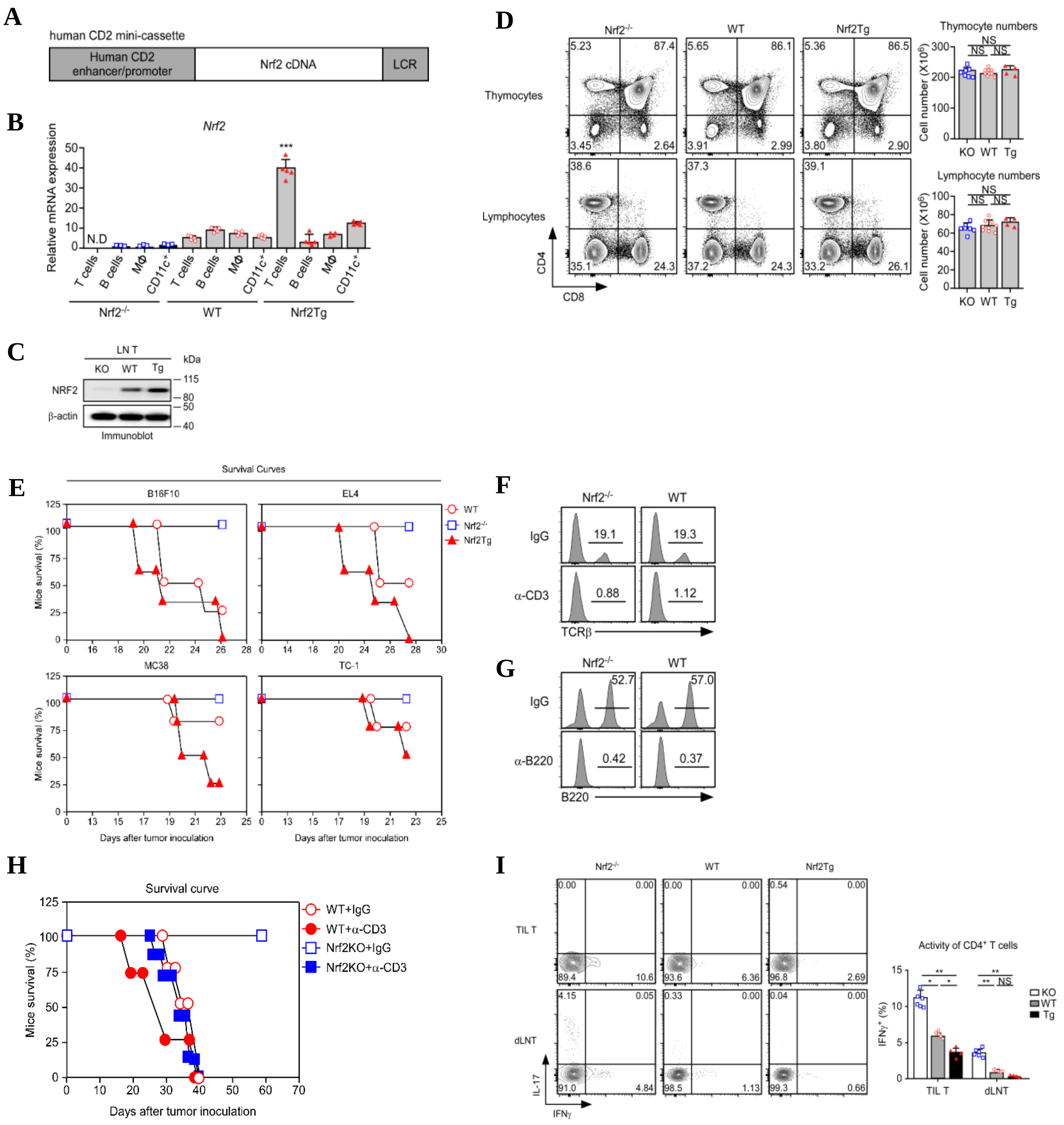
A
human CD2 mini-cassette
Human CD2 enhancer/promoter
Nrf2 cDNA
LCR
B
Nrf2
Relative mRNA expression
N.D
***
T cells
B cells
MΦ
CD11c+
Nrf2-/-
WT
Nrf2Tg
C
LN T
KO
WT
Tg
kDa
NRF2
β-actin
115
80
50
40
Immunoblot
D
Nrf2-/-
WT
Nrf2Tg
Thymocytes
Lymphocytes
CD4
CD8
5.23
87.4
3.45
2.64
5.65
86.1
3.91
2.99
5.36
86.5
3.80
2.90
38.6
35.1
24.3
37.3
37.2
24.3
39.1
33.2
26.1
Thymocyte numbers
Lymphocyte numbers
Cell number (X10^6)
NS
KO
WT
Tg
E
Survival Curves
B16F10
EL4
MC38
TC-1
WT
Nrf2-/-
Nrf2Tg
Mice survival (%)
Days after tumor inoculation
F
Nrf2-/-
WT
IgG
α-CD3
19.1
19.3
0.88
1.12
TCRβ
G
Nrf2-/-
WT
IgG
α-B220
52.7
57.0
0.42
0.37
B220
H
Survival curve
WT+IgG
WT+α-CD3
Nrf2KO+IgG
Nrf2KO+α-CD3
Mice survival (%)
Days after tumor inoculation
I
Nrf2-/-
WT
Nrf2Tg
TIL T
dLNT
IL-17
IFNγ
0.00
0.00
89.4
10.6
0.00
0.00
93.6
6.36
0.54
0.00
96.8
2.69
4.15
0.05
91.0
4.84
0.33
0.00
98.5
1.13
0.04
0.00
99.3
0.66
Activity of CD4+ T cells
IFNγ+ (%)
KO
WT
Tg
TIL T
dLNT
NS

Figure S2. Nrf2-deficient T cells promote tumor regression and effector functions.

(**A**) Scheme for Nrf2 construction. Generation of hCD2 mini-cassette-driven Nrf2 transgene. (**B**) *Nrf2* mRNA levels in immune cell subtypes (T cells, B cells, macrophages, and CD11c^{+}) from WT, Nrf2$^{-/-}$, and Nrf2Tg mice. The data represent the summary of three independent experiments (means ± SEM). N.D (nondetectable). (**C**) NRF2 protein levels in T cells from WT, Nrf2$^{-/-}$, and Nrf2Tg mice. β-actin was used as the loading control. The blot is representative of three independent experiments. (**D**) Contour plots showing CD4/CD8 profiles of total thymocytes and lymphocytes. The bar-graph presents representative of five independent experiments analysing 10 WT, 8 Nrf2$^{-/-}$, and 4 Nrf2Tg mice. (**E**) Survival rate of Nrf2$^{-/-}$, WT, and Nrf2Tg mice (n≥4 mice/group) injected s.c. with B16F10 melanoma, EL4 lymphoma, MC38 colon carcinoma, or TC-1 lung carcinoma cells. (**F** and **G**) Confirmation of T cell depletion using α-CD3 antibody (**F**) and B cell depletion using α-B220 antibody by blood staining (**G**). Histograms are representative of three independent experiments (n ≥4 mice/group). (**H**) Survival of Nrf2$^{-/-}$ and WT mice (n ≥ 4 mice/group) injected s.c. with EL4 lymphoma cells that received intraperitoneal injections of either α-CD3 or isotype control IgG once every five days. Results were pooled from three independent experiments. (**I**) IFNγ expression in CD4^{+} TIL T from Nrf2$^{-/-}$, WT, and Nrf2Tg tumor-bearing mice. TIL T cells and dLNT cells were stimulated for 4 hr with PMA/Ionomycin and assessed for IFNγ expression by intracellular staining. The IFNγ profile is representative of five independent experiments (left). The bar-graph depicts the percent (%) of IFNγ-producing T cells (right). Error bars show means ± SEM of five independent experiments (*p <0.05; **p <0.01; ***p<0.001 and NS, not significant).

Figure S3

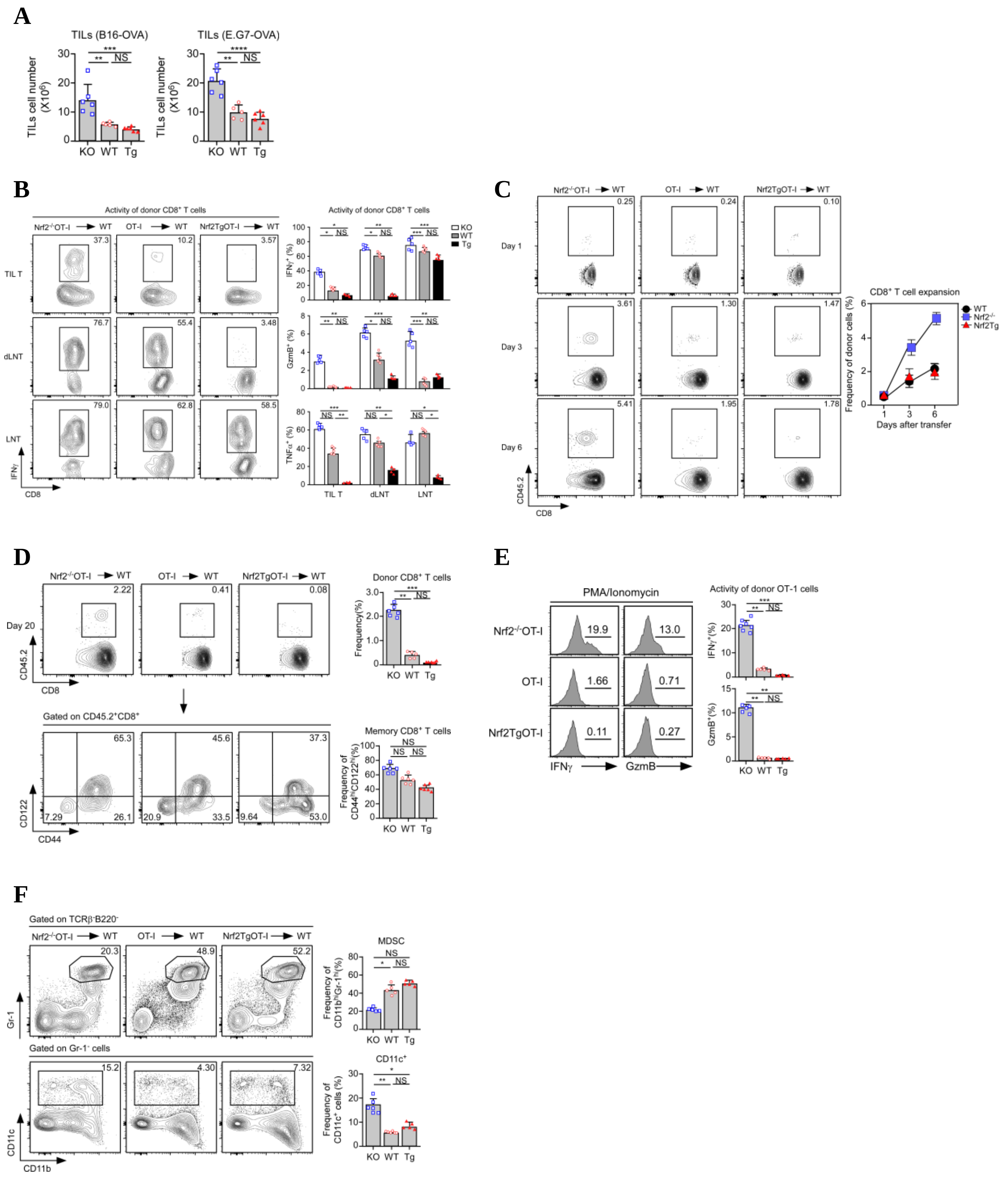
A
TILs (B16-OVA)
TILs (E.G7-OVA)
TILs cell number (X10⁶)
KO WT Tg
B
Activity of donor CD8⁺ T cells
TIL T
dLNT
LNT
IFNγ
CD8
C
Day 1
Day 3
Day 6
CD45.2
CD8
CD8⁺ T cell expansion
Frequency of donor cells (%)
Days after transfer
D
Day 20
Donor CD8⁺ T cells
Gated on CD45.2⁺CD8⁺
Memory CD8⁺ T cells
CD122
CD44
E
PMA/Ionomycin
Activity of donor OT-1 cells
IFNγ
GzmB
F
Gated on TCRβ⁻B220⁻
MDSC
Gated on Gr-1⁻ cells
CD11c⁺
Gr-1
CD11c
CD11b

Figure S3. Nrf2 attenuated anti-tumor responses of $CD8^+$ T cells.

(**A**) Total TIL cells were summarized in the tumor 23 days after tumor injection. The bar graph summarizes the TIL cell numbers in the tumor. Data are represented by three independent experiments (n ≥5 mice/group; means ± SEM). (**B**) TI T cells, dLNT, and LNT, isolated 20 days after E.G7-OVA challenge and were stimulated with $OVA_{257–264}$ for 16hr. IFNγ and GzmB expression was analysed in donor OT-I cells using intracellular staining. Contour plots are representative of four independent experiments (n ≥5 mice/group; left). The bar-graph represents the summary of four independent experiments (n ≥5 mice/group; means ± SEM; right). (**C**) Donor OT-I cells were identified in the blood collected on days 1, 3, and 6 after adoptive transfer. Contour plots are representative of two independent experiments (n ≥5 mice/group, left). The graph presents the summary of two independent experiments (n ≥5 mice/group; means ± SEM; right). (**D**) Maintenance of donor OT-I cells in the spleen 20 days after E.G7-OVA challenge. CD8 vs CD45.2 profiles of $CD4^-TCR\beta^+$ gated splenocytes (top) and CD44 vs CD122 profiles of donor OT-I cells (bottom). Contour plots are representative of two independent experiments (n ≥5 mice/group; left). The bar graph presents the summary of two independent experiments (n ≥5 mice/group; means ± SEM; right). (**E**) Splenocytes isolated 20 days after E.G7-OVA challenge were stimulated with PMA/Ionomycin and IFNγ, and GzmB expression was assessed in donor OT-I cells using intracellular staining. Histograms are representative of two independent experiments (n ≥5 mice/group; left). The bar-graph presents the summary of two independent experiments (n ≥5 mice/group; means ± SEM; right). (**F**) The percentages of $Gr\text{-}1^{hi}CD11b^{hi}$MDSC subsets in spleen. The percentages of $Gr\text{-}1^{hi}CD11b^{hi}$ MDSC subsets and the population of $CD11c^+$ DCs was analysed in gated on $TCR\beta^-B220^-$ cells and in gated $Gr\text{-}1^-$ cells, respectively. Contour plots are representative of two independent experiments (n ≥5 mice/group; left). The bar graph presents the summary of two independent experiments (n ≥5 mice/group; means ± SEM; right). (*p <0.05; **p <0.01; ***p<0.001, ****p<0.0001 and NS, not significant).

Figure S4

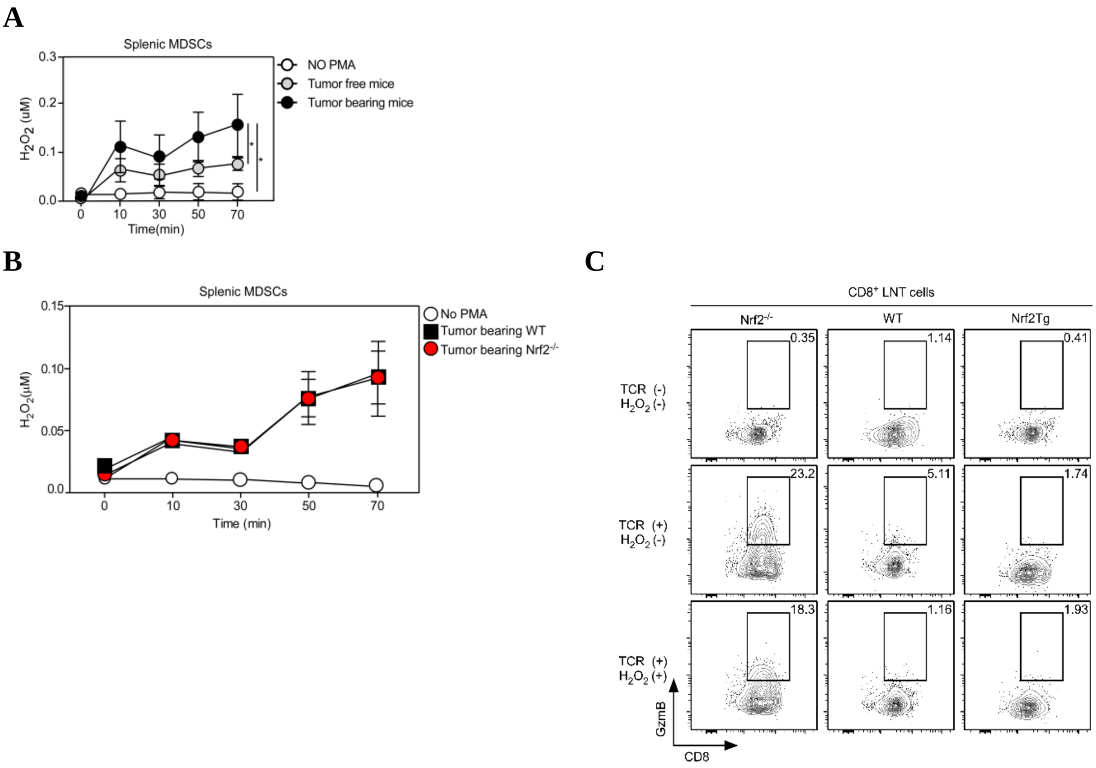

Figure S4. The MDSC-ROS-Nrf2 axis regulates $CD8^+$ T cell function.

(**A**) MDSCs were sorted from spleen of tumor (EL4) bearing or WT (tumor free) mice and were stimulated with PMA or medium control. H_2O_2 production was detected over time with Amplex Red reagent treatment. The results summarise three independent experiments. (**B**) Gr-1^{hi}CD11b^{hi} splenic MDSCs from WT and Nrf$2^{-/-}$ mice with B16F10 melanoma were incubated with Amplex Red reagent and stimulated with PMA, and H_2O_2 production was assessed at the indicated times. The results summarise three independent experiments. (**C**) Naïve T cells were stimulated with α-CD3/α-CD28 (0.1 μg/ml) for 16 hr and then activated T cells were incubated with 600 nM H_2O_2 for 12 hr. Intracellular GzmB were analysed. Contour plots are representative of five independent experiments.

Figure S5

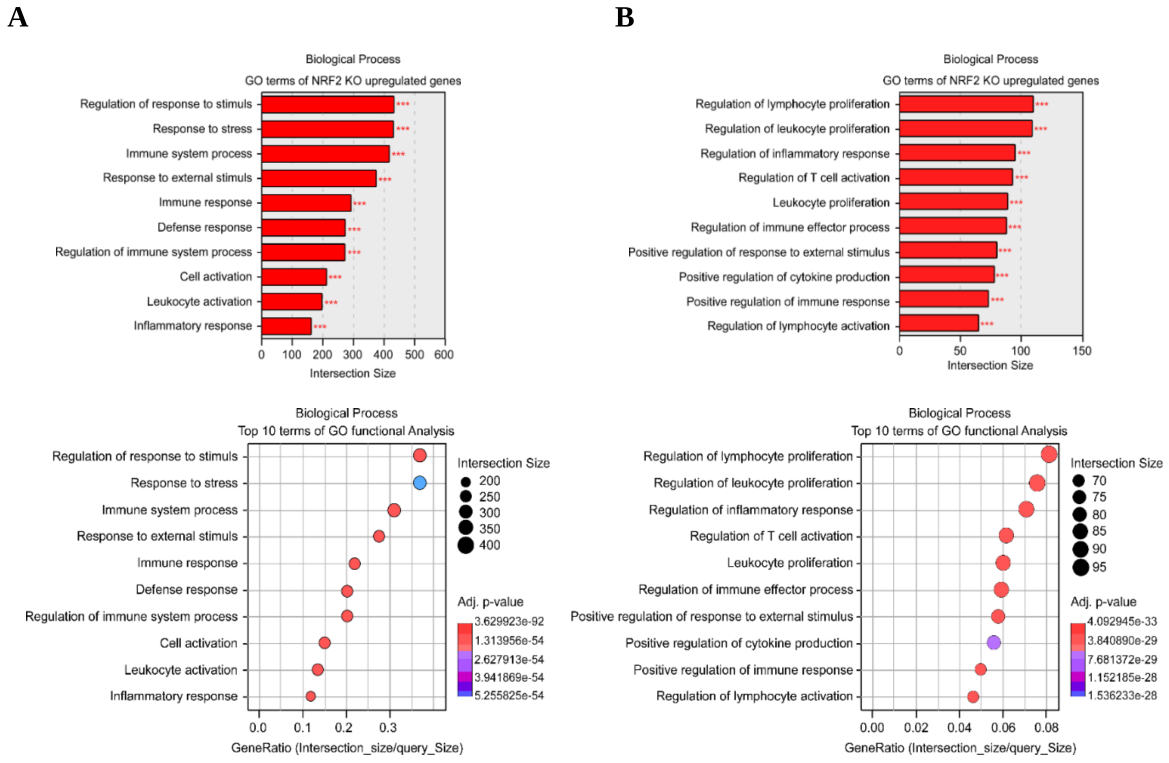

Figure S5. Gene expression profiles in TI Nrf2$^{-/-}$OT-I cells.

(**A** and **B**) Top 10 GO terms of upregulated genes in Nrf2-/-OT-I groups compared with WTOT-I groups from E.G7-OVA-tumor-bearing mice. (**A**) Top 10 GO terms of immune response-related genes in Nrf2-/-OT-I groups compared with WTOT-I group. (**B**) Top 10 GO terms of T cell activation-associated pathways in Nrf2-/-OT-I groups compared with WTOT-I group.

Figure S6

A

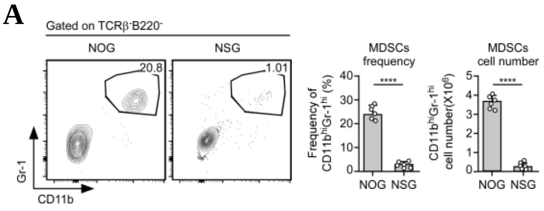

B

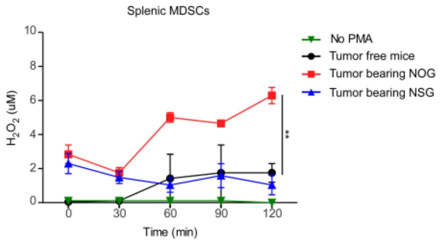

C

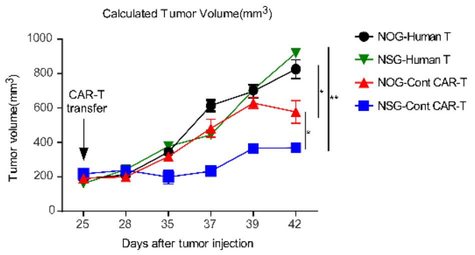

D

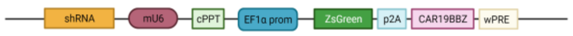

E

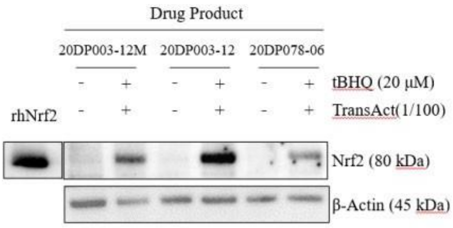

NRF2 Knockdown		NRF2 Intensity	Actin Intensity	NRF2/Actin	Relative Expression
Sample	20DP003-12M	2,636,324	3,116,490	0.85	0.74
	20DP003-12	5,671,235	4,984,182	1.14	1.00
	20DP078-06	1,509,927	3,868,104	0.39	0.34

~66% knockdown

F

sample		MOCK	shGFP	shNrf2
Viability(%)		84.2%	94.1%	88.7%
Viable T cell		100%	100%	100%
Viable CAR-T cell			97.9%	96.9%
T cell	CD4	18.0%	84.6%	91.7%
	CD8	71.2%	13.6%	7.0%
T cell subtyping	Naïve T cell	6.7%	5.3%	3.5%
	Central Memory T cell	15.5%	20.8%	26.2%
	Effector Memory T cell	38.4%	40.2%	50.1%
	Effector T cell	39.4%	33.7%	20.3%
CD4 T cell subtyping	Naïve T cell	5.0%	4.2%	3.0%
	Central Memory T cell	28.6%	23.6%	28.1%
	Effector Memory T cell	43.1%	44.4%	52.1%
	Effector T cell	23.3%	27.9%	16.9%
CD8 T cell subtyping	Naïve T cell	6.8%	12.1%	9.8%
	Central Memory T cell	11.8%	5.3%	4.9%
	Effector Memory T cell	34.9%	11.2%	18.4%
	Effector T cell	46.4%	71.4%	67.0%
Lymphocyte subtyping	T cell	100%	100%	100%
	B cell	0.0%	0.0%	0.0%
	NK cell	0.0%	0.0%	0.0%
	Monocyte	0.0%	0.0%	0.0%

Figure S6. Establishment and characterization of NRF2-modified human CD19-CAR-T cells. (**A**) The frequency of MDSCs in tumor bearing NSG and NOG mice. TILs were isolated from NSG and NOG mice with s.c. injected IM-9 tumor and CD11b vs Gr-1 profiles were analysed by gating on $TCR\beta^-$ $CD45R^-$ TILs. Contour plots are representative of two independent experiments (n =6 /group). The bar-graph presents the summary of two independent experiments (right, mean ± SEM). (**B**) MDSCs ($CD11b^{hi}Gr\text{-}1^{hi}$) isolated from tumor bearing NOG or NSG mice were stimulated with PMA and assessed for H_2O_2 production over time. Graphs are representative of two independent experiments (n=6 /group). (**C**) The anti-tumor efficacy of CAR-T cells in NSG and NOG mice. Human T or CAR-T cells were transferred to IM-9 tumor-bearing either NSG or NOG mice and tumor growth was monitored every 3-7 days. The results represent the summary of two independent experiments (n=6 /group). *p <0.05; **p <0.01; ***p<0.001, and NS, not significant. (**D**) Scheme of Nrf2 knockdown (Nrf2KD) CAR construction. (**E**) The efficiency of CAR-T cell transduction was confirmed by western blotting. The expression of the target proteins was normalized to that of β-actin. Knockdown efficiency = {(5,671,235/4,984,182)/ (5,671,235/4,984,182)-(1,509,927/3,868,104)/(5,671,235/4,984,182)}* 100 (%). The band Intensity software was BioRad ImageLab 6.0. The samples included: 20DP003-12M was Mock T cells, 20DP003-12 was Cont-CAR-T cells, and 20DP078-06 was Nrf2 KD-CAR-T cells. (**f**) Nrf2KD and control CAR-T cells were analyzed for T and CAR-T cell viability; lymphocyte subtypes including T, B, NK, and monocytes; and TN, TCM, and TEM cells within the $CD4^+$ and $CD8^+$ T cell subsets.

Figure S7

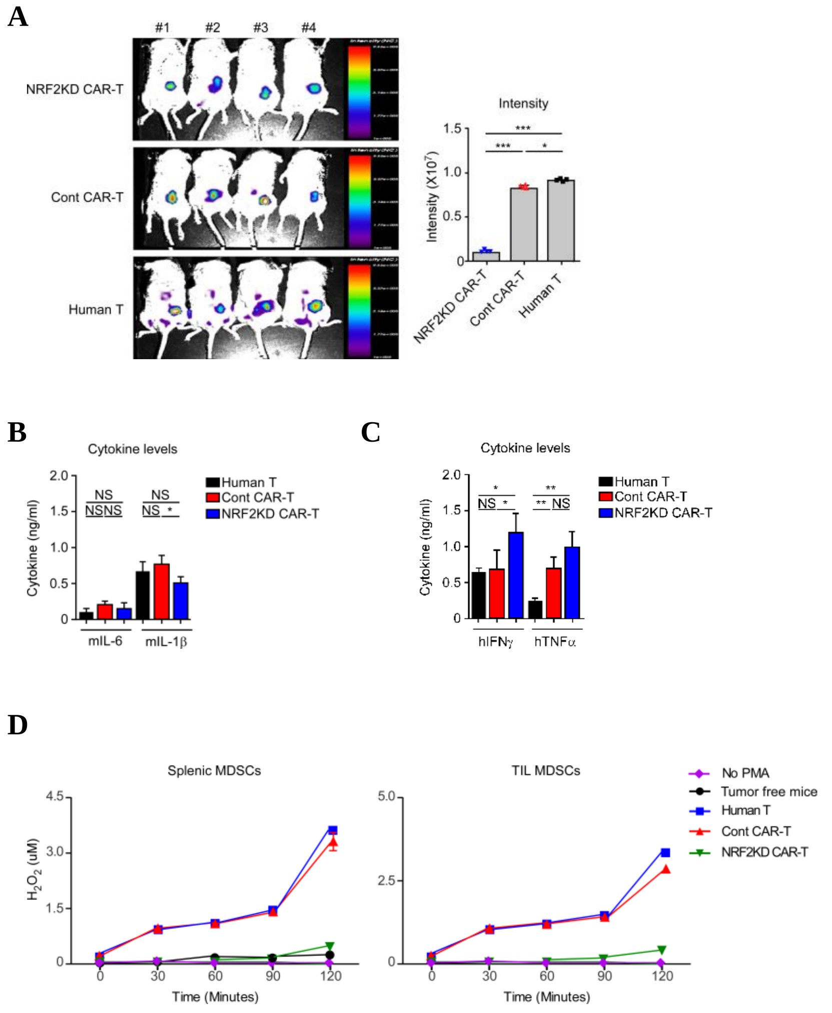

Figure S7. Nrf2 knockdown effect in CAR-T cell efficacy and ROS production by MDSCs in IM-9-tumor-bearing mice.

(**A**) NOG mice were subcutaneously inoculated with 10 × 10^6 IM-9 zsgreen cells. When the tumors reached an average volume of 250–300 mm^3 (day 27), the mice were infused with 1.5 × 10^6 control CAR-T, Nrf2KD CAR-T and control human T cells. Dorsal bioluminescence imaging of four representative mice bearing IM9-zsgreen tumors from each indicated group. (**B-C**) Serums were harvested on 17 days after CAR-T cell infusion and assessed for murine IL-6, murine IL-1β, human IFNγ and TNFα using ELISA. (Human T: n=3, Cont CAR-T: n=4, NRF2KD-CAR-T: n=5) *p <0.05; **p <0.01; ***p<0.001, and NS, not significant by two-tailed Student's t-tests. (**D**) ROS productivity

comparison of MDSC in the spleen (left) and TILs (right) from indicated mice groups. MDSCs isolated from IM-9-bearing NOG mice were stimulated with PMA or medium control. H_2O_2 production was detected over time with Amplex Red reagent treatment. The results summarise three independent experiments.

REFERENCES

1. Tirosh I, Izar B, Prakadan SM, Wadsworth MH, Treacy D, Trombetta JJ, Rotem A, Rodman C, Lian C, Murphy G, et al. (2016) Dissecting the multicellular ecosystem of metastatic melanoma by single-cell RNA-seq. Science (New York, NY).*352*, 189-96. http://doi.org/10.1126/science.aad0501.
2. Wherry EJ, Ha SJ, Kaech SM, Haining WN, Sarkar S, Kalia V, Subramaniam S, Blattman JN, Barber DL, and Ahmed R. (2007) Molecular signature of CD8+ T cell exhaustion during chronic viral infection. Immunity.*27*, 670-84. http://doi.org/10.1016/j.immuni.2007.09.006.
3. Baitsch L, Baumgaertner P, Devêvre E, Raghav SK, Legat A, Barba L, Wieckowski S, Bouzourene H, Deplancke B, Romero P, et al. (2011) Exhaustion of tumor-specific $CD8^+$ T cells in metastases from melanoma patients. J Clin Invest.*121*, 2350-60. http://doi.org/10.1172/jci46102.
